# A dopamine gradient controls access to distributed working memory in the large-scale monkey cortex

**DOI:** 10.1016/j.neuron.2021.08.024

**Published:** 2021-09-17

**Authors:** Sean Froudist-Walsh, Daniel P. Bliss, Xingyu Ding, Lucija Rapan, Meiqi Niu, Kenneth Knoblauch, Karl Zilles, Henry Kennedy, Nicola Palomero-Gallagher, Xiao-Jing Wang

**Affiliations:** 1 Center for Neural Science, New York University, New York, NY 10003, USA; 2 Research Centre Jülich, INM-1, Jülich, Germany; 3 INSERM U846, Stem Cell & Brain Research Institute, 69500 Bron, France; 4 Université de Lyon, Université Lyon I, 69003 Lyon, France; 5 Institute of Neuroscience, State Key Laboratory of Neuroscience, Chinese Academy of Sciences (CAS), Key Laboratory of Primate Neurobiology CAS, Shanghai, China; 6 C. & O. Vogt Institute for Brain Research, Heinrich-Heine-University, 40225 Düsseldorf, Germany; 7 Senior author; 8 Deceased April 26, 2020; 9 Lead contact

## Abstract

Dopamine is required for working memory, but how it modulates the large-scale cortex is unknown. Here, we report that dopamine receptor density per neuron, measured by autoradiography, displays a macroscopic gradient along the macaque cortical hierarchy. This gradient is incorporated in a connectome-based large-scale cortex model endowed with multiple neuron types. The model captures an inverted U-shaped dependence of working memory on dopamine and spatial patterns of persistent activity observed in over 90 experimental studies. Moreover, we show that dopamine is crucial for filtering out irrelevant stimuli by enhancing inhibition from dendrite-targeting interneurons. Our model revealed that an activity-silent memory trace can be realized by facilitation of inter-areal connections and that adjusting cortical dopamine induces a switch from this internal memory state to distributed persistent activity. Our work represents a cross-level understanding from molecules and cell types to recurrent circuit dynamics underlying a core cognitive function distributed across the primate cortex.

## In brief

Little is known about how dopamine outside of the prefrontal cortex affects working memory. Froudist-Walsh et al. identify a gradient of dopamine receptors in the macaque cortex and use this to build a large-scale computational cortex model. A gradient of cortical dopamine modulation provides a parsimonious explanation for diverse findings in the literature.

## Graphical Abstract

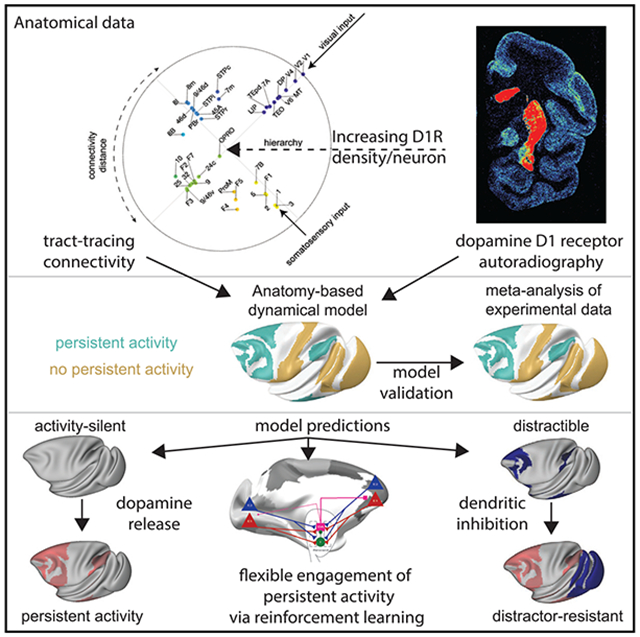

## INTRODUCTION

Our ability to think through difficult problems without distraction is a hallmark of cognition. When faced with a constant stream of information, we must keep certain information in mind and protect it from distraction. For instance, when at the supermarket looking for your favorite butter, it is important to keep in mind its distinctive golden packaging and not be distracted by the many other dairy products. This brain function is called working memory. Working memory often engages persistent neural activity that is specific to the information that must be remembered. This mnemonic activity is sustained internally across multiple cortical and subcortical areas in the absence of external stimulation (Funahashi et al., 1989; Fuster and Alexander, 1971; Guo et al., 2017; Leavitt et al., 2017; Mejias and Wang, 2021; Mendoza-Halliday et al., 2014; Murray et al., 2017; Romo et al., 1999; Romo and Salinas, 2003; Vergara et al., 2016; Wang, 2001; Zhang et al., 2019).

Working memory and the prefrontal cortex are under the influence of monoaminergic modulation (Goldman-Rakic, 1995; Robbins and Arnsten, 2009). In fact, depletion of dopamine from the prefrontal cortex and complete ablation of the prefrontal cortex cause similar working memory deficits (Brozoski et al., 1979). Dopamine modulates cortical activity through its receptors. D1 receptors are the most densely expressed dopamine receptor type in the cortex. Prefrontal neuron activity during working memory depends on precise levels of activation of D1 receptors, with too little or too much D1 stimulation disrupting delay period activity (Vijayraghavan et al., 2007; Wang et al., 2019). However, the density of D1 receptors is known only for relatively small sections of the monkey cortex (Goldman-Rakic et al., 1990; Impieri et al., 2019; Lidow et al., 1991; Niu et al., 2020; Richfield et al., 1989). Because of the shortage of areas analyzed across studies, it is not clear whether the variation in D1 receptor densities across cortical areas represents random heterogeneity or a systematic gradient of cortical dopamine modulation.

Dopamine receptors are also expressed differently across different types of inhibitory neurons (Mueller et al., 2018, 2020). Distinct inhibitory cell types primarily focus their inhibition on the dendrites or somata of pyramidal cells or on other inhibitory neurons (Jiang et al., 2015; Tremblay et al., 2016). Through its differing effects on distinct interneurons, dopamine decreases inhibition to the somata of pyramidal cells and increases inhibition to the dendrites (Gao et al., 2003). An early theoretical study proposed that inhibition targeted more strongly toward the dendrites and away from the somata of pyramidal cells could increase the resistance of working memory to distraction (Wang et al., 2004a). The functional significance of dopamine’s differential effects on distinct inhibitory neuron types has not yet been investigated.

In this work, we tackled two open questions. First, how does dopamine modulate distributed working memory across a multi-regional large-scale cortical system? Second, in light of the emphasis on cell types in modern cortical physiology, does dopamine contribute to robust working memory against distractors by virtue of differential effects on different neuron classes? To address these questions, we performed quantitative mapping of dopamine D1 receptor densities across 109 cortical areas using *in vitro* autoradiography and constructed a large-scale computational model of the macaque cortex that is capable of performing working memory tasks. The model is built using retrograde tract-tracing connectivity data and incorporates gradients of D1 receptors and excitatory synapses. Moreover, to our knowledge, this is the first large-scale cortex model endowed with three subtypes of inhibitory neurons. Our results suggest that firing of dopamine neurons can engage distractor-resistant, stimulus-selective, sustained activity across multiple brain regions in response to behaviorally relevant stimuli. Furthermore, we extend, from a local area to the multi-regional cortex, an activity-silent mechanism that has been proposed for certain forms of short-term memory trace without persistent activity (Mongillo et al., 2008; Rose et al., 2016; Wolff et al., 2017). We found that this scenario relies principally on short-term facilitation of inter-areal connections but fails to resist distractors. Enhanced dopamine modulation can convert an internal memory trace to an active persistent activity state needed to filter out distractors. Therefore, our findings contribute to resolving the current debate about the two contrasting scenarios that contribute to working memory (Constantinidis et al., 2018; Lundqvist et al., 2018; Watanabe and Funahashi, 2014) and under what conditions each mechanism is implemented (Barbosa et al., 2020; Masse et al., 2019; Trübutschek et al., 2019).

## RESULTS

### A hierarchical gradient of dopamine D1 receptors per neuron across the monkey cortex

**Figure 1.**
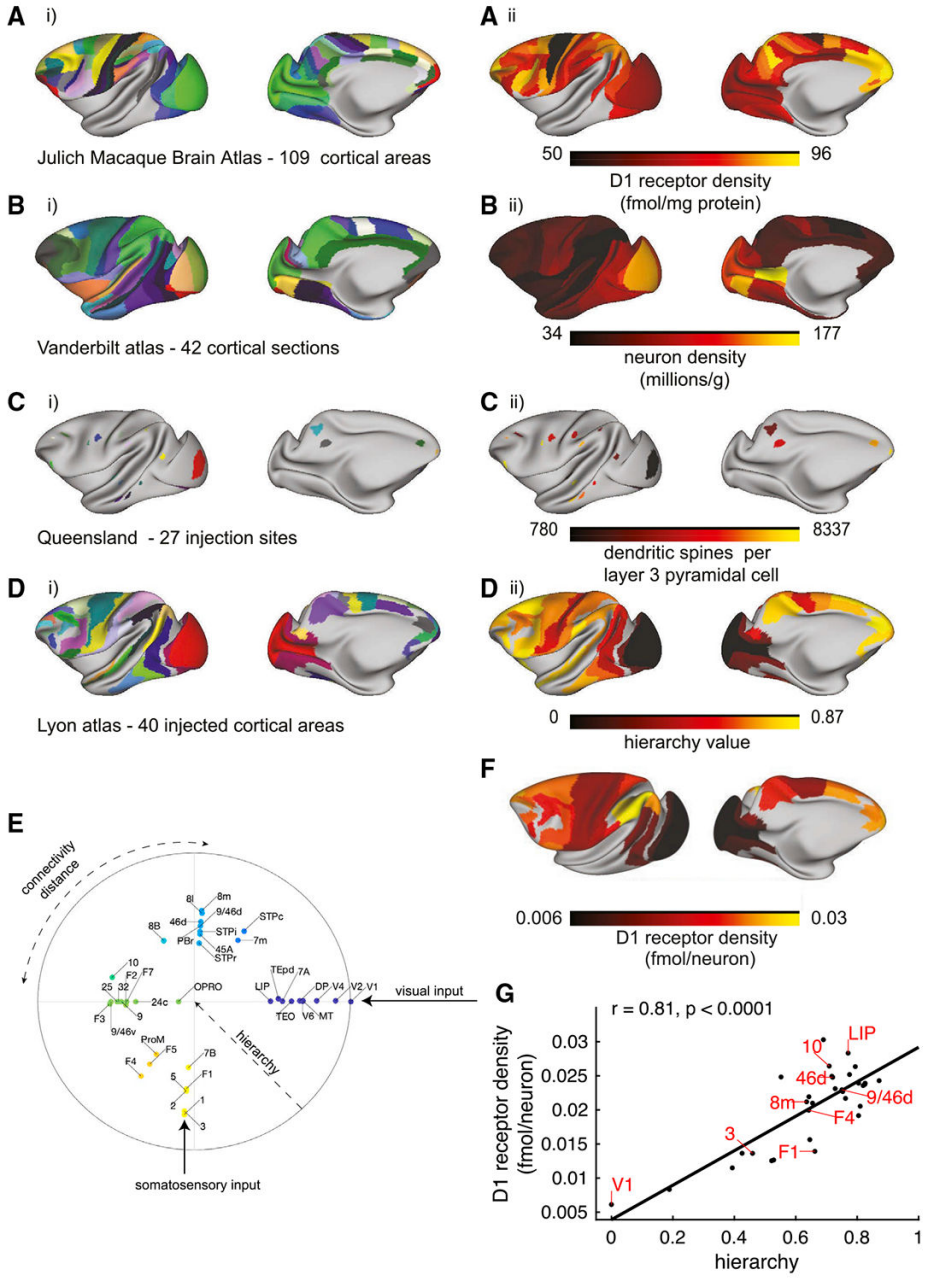
A gradient of dopamine D1 receptors per neuron across the monkey cortex (A) i: 109 cortical regions of the Julich Macaque Brain Atlas, identified by receptor and cytoarchitecture. ii:D1 receptor density. The receptor density shown here does not take into account differences in neuron density across areas. (B) i: Collins et al., (2010) divided the macaque cortex into 42 slabs of tissue, here mapped on to the Yerkes19 surface. ii: neuron density across the cortex. (C) i: injection sites for the studies of dendritic spine density by Elston (2007). ii: number of dendritic spines on the basal dendrites of layer III pyramidal cells. (D) i: 40 injected areas in the retrograde tract-tracing database of Markov et al. (2014b). ii: cortical hierarchy. (E) Circular embedding of the cortical hierarchical connectivity structure. Radial distance to the center represents the hierarchical position of the area, with the areas lowest in the hierarchy closest to the edge. Angular distance between areas represents the inverse of connectivity strength (fraction of labeled neurons - FLN), so that areas that are plotted at similar angles are more strongly connected to each other. Colors represent the angle on the circle. Clear visual and somatosensory hierarchies emerge from this circular embedding of the connectivity data (highlighted with arrows). Association areas lie at angles off the main visual and somatosensory hierarchies. (F) The density of D1 receptors divided by neuron density. Regions that have not yet been measured are shown in gray. (G) There was a strong positive correlation between the D1 receptor density per neuron and the cortical hierarchy. The spatially corrected p value is the fraction of randomly generated surrogate maps with spatial smoothness matched to the hierarchy map that show a stronger Pearson correlation (negative or positive) with the D1 receptor map than the hierarchy map itself. See also Figures S1 and S2.

We first analyzed D1 and D2 receptor distribution patterns throughout the macaque brain using *in vitro* receptor autoradiography (Figure S1). Autoradiography enables quantification of endogenous receptors in the cell membrane through use of radioactive ligands (Niu et al., 2020; Palomero-Gallagher and Zilles, 2018; Rapan et al., 2021). The highest densities (in fmol/mg protein) of both receptor types were found in the basal ganglia, with the caudate nucleus (D1, 298 ± 28; D2, 188 ± 30) and putamen (D1, 273 ± 40; D2, 203 ± 37) presenting considerably higher values than the internal (D1, 97 ± 34; D2, 22 ± 12) or external (D1, 55 ± 16; D2, 30 ± 11) subdivisions of the *globus pallidus*. Raw cortical D1 receptor densities ranged from 49 ± 13 fmol/mg protein in area 4a of the primary motor cortex to 101 ± 35 fmol/mg protein in orbitofrontal area 11l (Figure 1A). The density of the D2 receptor in the cortex is so low that it is not detectable with the method used here.

To compare the gradient of D1 receptors with other known gradients of anatomical organization in the monkey cortex, we carefully mapped the receptor data (Figure 1A) as well as data on neuronal density (Figure 1B; Collins et al., 2010) and spine count (Figure 1C; Elston, 2007) onto the Yerkes19 common cortical template, to which anatomical tract tracing data (Figure 1D, i) has been mapped previously (Donahue et al., 2016). Here we include retrograde tracing data from 40 regions, quantified using the same protocol as in previous publications (Markov et al., 2014b). This expands the number of injected cortical areas by 33%, with connections to areas 1, 3, V6, F4, F3, 25, 32, 9, 45A, and OPRO (orbital proisocortex) now included in the database (downloadable from core-nets.org). We estimated the cortical hierarchy using laminar connectivity data (Figure 1D, ii; STAR Methods; Markov et al., 2014a), expanding previous descriptions of the cortical hierarchy based on fewer regions (Markov et al., 2014a; Mejias et al., 2016). A one-dimensional hierarchy is probably an oversimplification of the cortical connectivity structure. Because we have connectivity data for two distinct sensory modalities, we also calculated a circular embedding of the connectivity data, with radial distance from the edge representing the hierarchical position and angular distance between points representing the inverse of their connectivity strength (Chaudhuri et al., 2015). In this circular representation, separate visual and somatosensory hierarchies can clearly be appreciated, with association regions falling at angles off the main sensory hierarchy axes (Figure 1E).

To facilitate functional interpretation, we divided D1 receptor density by neuron density (Collins et al., 2010) to allow estimation of the degree to which dopamine modulates individual neurons across the cortex. D1 receptor density per neuron peaked in the parietal and frontal cortex and was relatively low in the early sensory cortex (Figure 1F). There was a strong positive correlation between D1 receptor density per neuron and the cortical hierarchy (Figure 1G; r = 0.81). Because of spatial autocorrelation between cortical features (i.e., nearby parts of the cortex tend to have a similar anatomy), it is possible to detect spurious correlations between distinct features of brain anatomy. To account for this, we generated 10,000 surrogate maps with similar spatial autocorrelation to the hierarchy map (Burt et al., 2020). None of these surrogate maps were as strongly correlated with the D1 receptor density map as the hierarchy, giving a p value of less than 0.0001 for the D1 receptor-hierarchy correlation. There was no significant relationship between D1 receptor expression and whether a cortical area had a granular layer IV (Wilcoxon rank-sum Z = 0.39, p = 0.70) or to the degree of externopyramidalization (Kruskal-Wallis χ^2^ = 1.47, p = 0.48; Goulas et al., 2018; Sanides, 1962; Figure S2). This pattern of receptor expression suggests that dopamine principally modulates areas contributing to higher cognitive processing.

### A cortical circuit with three types of inhibitory neurons modulated by dopamine

**Figure 2.**
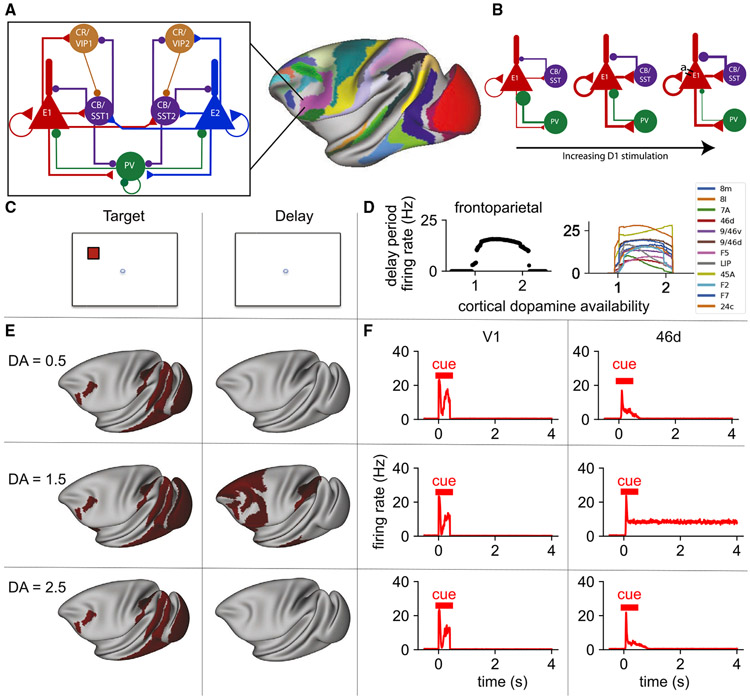
An inverted U relationship between D1 receptor stimulation and distributed frontoparietal delay period activity (A) Left: local circuit design. The circuit contains two populations of excitatory cells (red and blue), each selective to a particular spatial location. The cell bodies (triangles) and dendrites (cylinders) are modeled as separate compartments. PV (green), CB/SST (purple), and CR/VIP (light brown) cells have characteristic connectivity patterns. Right: the local circuit is placed at each of 40 cortical locations (various colors). Cortical areas differ in (1) inter-areal connections, (2) spine count, and (3) dopamine D1 receptor density. (B) Stimulation of D1 receptors affects the cortical circuit via (1) an increase in inhibition targeting the dendrites with a corresponding decrease in inhibition to the somata of pyramidal cells, (2) an increase in NMDA-dependent excitatory transmission for low to medium levels of stimulation, and (3) increasing adaptation for high levels of stimulation. (C) Structure of the task. The cortical network was presented with a stimulus it had to maintain through a delay period. (D) Left: mean firing rate in the frontoparietal network at the end of the delay period for different levels of dopamine release. Right: mean delay period activity of cortical areas as a function of dopamine release. All areas shown display persistent activity in experiments (Leavitt et al., 2017). (E) Activity is shown across the cortex at different stages in the working memory task (left to right), with increasing levels of dopamine release (from top to bottom). Red represents activity in the excitatory population sensitive to the target stimulus. Very low or very high levels of dopamine release resulted in reduced propagation of stimulus-related activity to frontal areas and a failure to engage persistent activity. Mid-level dopamine release enables distributed persistent activity. (F) Time courses of activity in selected cortical areas. The horizontal bars indicate the timing of cue (red) input to area V1. DA, cortical dopamine availability. See also Figures S3 and S4 and Video S1.

We built a model of a local cortical circuit that contains pyramidal cells and three types of inhibitory neurons (Figure 2A). The cortical circuit is based on a disinhibitory motif that was originally predicted theoretically (Wang et al., 2004a), with details of the connectivity structure chosen to reflect recent experimental findings (STAR Methods).

In our model, dopamine acted by increasing the synaptic strength of inhibition to the dendrite and reducing the synaptic strength of inhibition to the cell body of pyramidal cells (Figure 2B; Gao et al., 2003). In addition, dopamine increased the strength of transmission via N-methyl-D-aspartate (NMDA) receptors (Seamans et al., 2001). On the other hand, high stimulation of D1 receptors resulted in increased adaptation in excitatory cells (potentially an M-current, via KCNQ potassium channels; Arnsten et al., 2019), mimicking the net inhibitory effect of high concentrations of D1 agonists.

### A large-scale model of the macaque cortex incorporating multiple macroscopic gradients

We then built a large-scale model of the macaque cortex. We placed the local circuit in each of the 40 cortical areas (Figure 2A, right). Properties of these local circuits varied across areas in the form of macroscopic gradients (Wang, 2020) of long-distance connectivity (set by tracing data), strength of excitation (set by the spine count), and modulation by D1 receptors (set by the receptor autoradiography data). We defined the connections between areas using the quantitative retrograde tract-tracing data. In the model, inter-areal connections are excitatory and target the dendrites of pyramidal cells (Petreanu et al., 2009). Inter-areal excitatory connections also target calretinin (CR)/vaso-active intestinal peptide (VIP) cells to a greater degree than parvalbumin (PV) or calbindin (CB)/somatostatin (SST) cells (Lee et al., 2013; Wall et al., 2016). The frontal eye fields (FEF) have an unusually high density of CR (here CR/VIP) cells (Pouget et al., 2009). To account for this, we increased the proportion of inter-areal input to CR/VIP cells in FEF and reduced the strength of input to PV and CB/SST cells.

### An inverted U relationship between cortical D1 receptor stimulation and distributed working memory activity

We simulated the large-scale cortical model during performance of a working memory task (Figure 2C) with different levels of cortical dopamine availability. In simulations, stimulus-selective activity propagated from the visual cortex to the temporal, parietal, and frontal cortex. Activity in the visual cortex was relatively insensitive to dopamine (Figures 2E and 2F). Dopamine modulation had little to no effect on the initial peak of activity in early visual areas, but it did modulate the later peak of activity in these areas (Figure S3), consistent with a specific role of feedback connections in late visual activity (Self et al., 2012). In all cases, there was a strong transient response in visual areas prior to rapid return to baseline firing rates. This is similar to the response seen in neurons recorded from area V1 in behaving monkeys (van Vugt et al., 2018). We observed similar transient activity in somatosensory areas in response to stimulus input to the somatosensory cortex (Figure S4), as seen experimentally (Romo and Rossi-Pool, 2020). Delay period activity in a large network of prefrontal, lateral parietal, and temporal areas showed an inverted U relationship with dopamine levels (Figure 2D). A midrange level of dopamine release engaged a distributed pattern of persistent activity throughout these areas (Figures 2E and 2F), but release that was too low or too high only led to a transient response (Figure 2F). A similar pattern of delay period activity was observed following somatosensory input (Figure S4). The inverted U relationship between D1 receptor stimulation and working memory activity has been shown locally in the prefrontal cortex in experimental and computational studies (Brunel and Wang, 2001; Vijayraghavan et al., 2007) but has not been described previously throughout the distributed cortical system.

### Inter-areal connectivity determines the distributed working memory activity pattern

**Figure 3.**
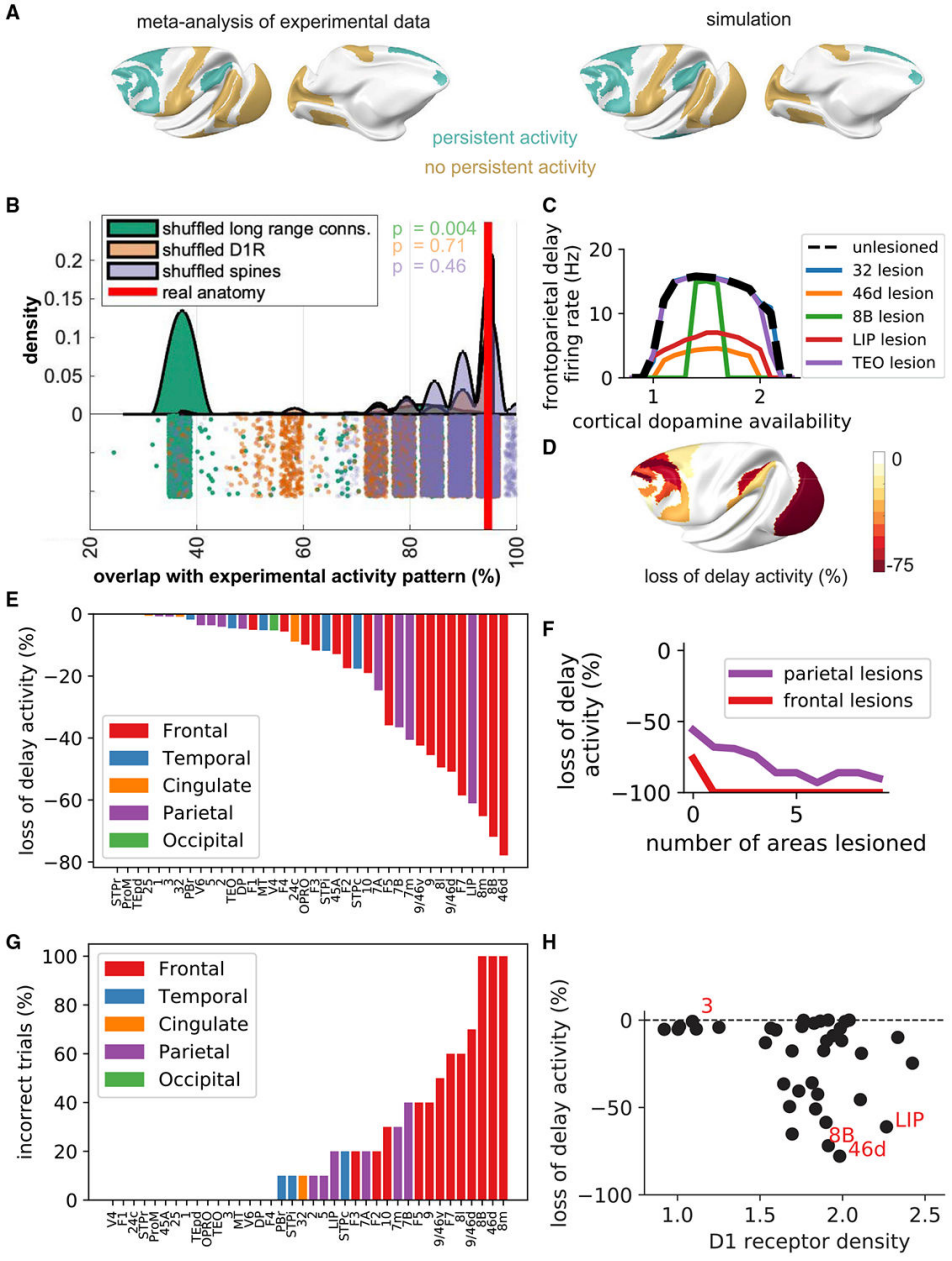
Inter-areal connectivity and D1 receptor density underlie working memory activity and performance (A) There is a strong overlap (18 of 19, 95%) between the pattern of persistent activity seen experimentally (Leavitt et al., 2017) and that predicted by the model. (B) The results of 10,000 simulations using shuffled inter-areal connections (green), 10,000 simulations using shuffled patterns of D1 receptor expression (orange), and 10,000 simulations using shuffled patterns of dendritic spine counts (purple). The position on the x axis denotes the overlap between the simulated delay activity pattern and the experimental activity pattern identified by Leavitt et al. (2017) for each simulation based on shuffled anatomical data. The red vertical line denotes the overlap between the simulation based on the real anatomy data and the experimental results. The bottom half of the image shows the results of individual simulations based on shuffled anatomical data. The top half of the image shows the densities. The pattern of inter-areal connections was the most important determinant of the working memory activity pattern. The p value is calculated as the fraction of simulations based on shuffled anatomical data that produce a delay activity pattern that overlaps with the experimental data as well as (or better than) the original simulation. (C) Lesions to areas such as 46d and LIP led to reduced delay-period firing across for all levels of dopamine release. Following some lesions (such as to area 8B), an optimal level of D1 receptor stimulation could restore close to normal working memory activity in the remaining network. (D) The level of disruption to distributed working memory activity following lesions to each area, quantified as the total loss of working memory activity in the frontoparietal network summed across all dopamine release levels. (E) The percent loss of delay period activity throughout the cortex following a lesion to each area. (F) The percent loss of delay period activity following progressively bigger lesions to frontal and parietal areas. (G) The percent of failed trials, across all dopamine levels, on a working memory task with a distractor following lesions to each cortical area. (H) Lesions to areas with a higher D1 receptor density tended to have a larger effect on working memory activity. D1R, D1 receptor density. See also Figure S5 and Tables S7 and S8.

We next compared the pattern of delay period activity in the model with delay period activity observed in over 90 electrophysiology studies (Leavitt et al., 2017). We chose model parameters that would produce persistent activity in the prefrontal cortex, but we did not fit the model to the experimental data. Of the 19 cortical areas in which such activity has been assessed during the delay period in at least three experimental studies, 18 were in agreement between the simulation and experimental results (χ^2^ = 15.03, *p* = 0.0001 Figure 3A). Overall, the experimentally observed persistent activity from numerous studies is reproduced, validating the model. This allows us to inspect the anatomical properties that underlie the distributed activity pattern and gain insight into the brain mechanisms that may produce it.

We repeated model simulations after shuffling the anatomical data. The delay period activity patterns for 30,000 simulations based on the shuffled anatomy were compared with the pattern observed experimentally. Ten thousand simulations were run using shuffled inter-areal connections, shuffled D1 receptor expression, and shuffled dendritic spine expression separately. The overlap between the experimental persistent activity pattern and the model persistent activity pattern was strongly dependent on the inter-areal connections (p = 0.0004) but not on the pattern of D1 receptors (p = 0.71) or dendritic spine count (p = 0.46) (Figure 3B). This analysis suggests that the edges between nodes in the network (i.e., the inter-areal connections) are important for defining the spatial pattern of delay period activity. Next we asked how the nodes themselves (i.e., individual cortical areas) contribute differentially to distributed working memory.

### Working memory deficits are most severe following lesions to prefrontal areas with high D1 receptor density

We next quantified the degree to which focal lesions to individual areas in the model disrupted persistent activity during the working memory task (without distractors). The effect depended on the lesioned area and the level of cortical dopamine (Figure 3C). Lesions to prefrontal and posterior parietal areas caused the greatest reductions in delay period firing rates (Figure 3D,E). Lesions to frontal areas caused a significantly greater reduction in delay period firing rates than lesions to parietal areas (Mann-Whitney *U* = 46.0, p = 0.027). We tested the effects of progressively larger lesions to the frontal and parietal cortex. To increase the size of the lesions, for each lobe we first lesioned the area that caused the biggest drop in delay activity when lesioned individually and then additionally lesioned the area that caused the second biggest drop and so on (frontal lesion 1: 46d, lesion 2: 46d+8B, lesion 3: 46d+8B+8 m etc.; parietal lesion 1: LIP, lesion 2: LIP+7m, lesion 3: LIP+7 m+7B. etc.). When lesioning two frontal regions, the mnemonic delay period activity was completely destroyed throughout the cortex, so the network was no longer able to perform the task. In contrast, progressively larger lesions of the parietal cortex caused only a gradual decrease in frontoparietal delay activity, and even when the entire parietal cortex was removed (10 areas), sufficient residual mnemonic delay period activity remained to allow the cue stimulus to be decoded (Figure 3F).

We subsequently addressed the ability of the model to maintain cue-specific delay period activity in the presence of distractors following precise lesioning of each cortical area. We analyzed trials across all levels of cortical dopamine availability. Lesions to three prefrontal areas (8m, 46d, and 8B), but not other areas, caused complete disruption of distractor-resistant working memory activity in all trials. Lesions to many other areas caused complete reduction of distractor-resistant working memory activity for some trials (corresponding to a particular dopamine range) but not others. The seven lesions causing the greatest disruption of working memory performance were in the frontal cortex (six prefrontal areas and premotor area F7; Figure 3G). The reduction in performance was significantly greater for lesions to frontal cortical areas than parietal areas (Mann-Whitney *U* = 48.5, p = 0.032). Our simulations thus suggest that (1) lesions to the prefrontal and posterior parietal cortex can cause a significant disruption of delay period activity, (2) frontal lesions have a greater effect on behavior than parietal lesions, and (3) smaller lesions, particularly to the prefrontal cortex, can significantly disrupt performance on more difficult working memory tasks, such as those with distractors. In contrast, larger lesions are required to disrupt performance on simple working memory tasks.

Lesions to area V1 and V2 led to complete loss of visual working memory activity (Figure 3D). However, this was because of the fact that a visual stimulus must go through area V1 to gain access to the working memory system. We confirmed this by showing that lesions to V1 and V2 had no effect on working memory when somatosensory stimuli were used (with stimulus presented to primary somatosensory area 3). In the somatosensory working memory task, lesions to early somatosensory areas and frontoparietal network areas caused memory deficits (Figure S5). This clearly separates early sensory areas, which are required for signal propagation to the working memory system, from core cross-modal working memory areas in the prefrontal and posterior parietal cortex.

D1 receptor density (F = 4.72, p = 0.036; Figure 3H) was the strongest anatomical predictor of the lesion effects, and adding hierarchy or spine count to the model did not significantly improve the fit. Thus, our model predicts that lesions to areas with a higher D1 receptor density are more likely to disrupt working memory activity. This prediction can be tested experimentally.

### Dopamine shifts between activity-silent and persistent activity modes of working memory

**Figure 4.**
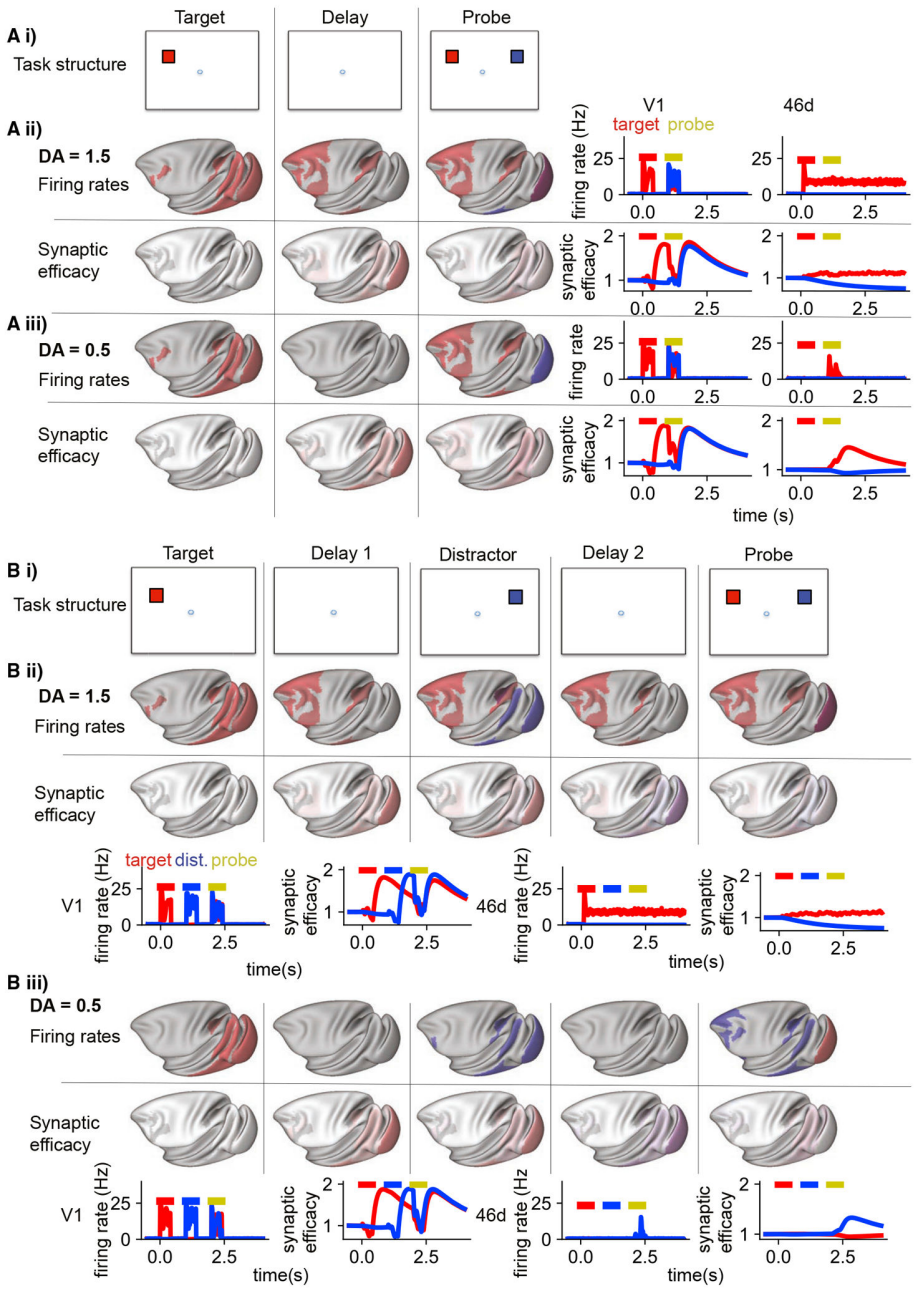
A dopamine-dependent shift between distractible activity-silent and distractor-resistant persistent activity states For a Figure360 author presentation of this figure, see https://doi.org/10.1016/j.neuron.2021.08.024. (A) i: task structure. A target stimulus was followed by a delay and a probe stimulus. ii: for mid-level dopamine release, activity relating to the target stimulus propagated from V1 through the hierarchy and was maintained in persistent activity throughout the frontoparietal network. Top: firing rates on the surface (left) and in selected areas (right). Bottom: synaptic efficacy. iii: for low-level dopamine release, activity (top) in response to the stimulus was transient in visual and some frontoparietal areas. There was no persistent activity through the delay period. However, in response to the probe stimulus, activity representing the original target stimulus was regenerated throughout the frontoparietal cortex. Bottom: the memory for the stimulus was stored as an increase in synaptic efficacy through the delay period, mostly in connections from sensory areas. (B) i: task structure. A target stimulus was followed by a delay period, a distractor, another delay period, and a probe stimulus. ii: for mid-level dopamine release, target-related activity was maintained in persistent activity throughout the frontoparietal network throughout the delay period through the distractor until the end of the trial. iii: for low-level dopamine release, frontoparietal activity related to the most recent stimulus (i.e., the distractor) was regenerated during this probe stimulus. See also Figure S6.

Recent experimental and modeling results show that some delay tasks can be solved with little or no persistent activity (Mongillo et al., 2008; Rose et al., 2016; Watanabe and Funahashi, 2014; Wolff et al., 2017). This has spurred a debate about whether persistent activity or “activity-silent” mechanisms underlie working memory (Constantinidis et al., 2018; Lundqvist et al., 2018). Is dopamine modulation throughout the cortex relevant to this debate? We endowed the model with short-term plasticity to assess the possibility of activity-silent working memory in the large-scale network. Short-term plasticity was implemented at all synapses between excitatory cells (using the same parameters as Mongillo et al., 2008) and from excitatory to CB/SST cells. We investigated activity-silent representations by “pinging” the system with a neutral stimulus and reading out the activity generated in response, similar to the experimental protocol in Wolff et al. (2017) (Figure 4A, i). For optimal midlevels of dopamine release (Figure 4A, ii), the model generated persistent activity that was very similar to the network without short-term plasticity. The strong and distributed activation of the frontal and parietal cortex is reminiscent of the ignition response to consciously observed stimuli (van Vugt et al., 2018).

For low and high levels of dopamine release, there was no persistent activity (Figure 4A, iii). However, when we pinged the system with a neutral stimulus, activity relating to the target cue was generated transiently throughout the frontoparietal network (Figure 4A, iii), suggesting that a memory of the target stimulus was stored internally. During the delay period, the synaptic efficacy increased at connections between neurons coding for the target stimulus. Previous models of activity-silent short-term memory have focused on local synaptic changes in the prefrontal cortex (Mongillo et al., 2008). In our model, most of the increase in synaptic efficacy was in synaptic connections from neurons in sensory areas (Figure 4A, iii). We then restricted short-term synaptic plasticity to presynaptic neurons outside of the frontoparietal network. Pinging this system again resulted in activation of the target-related activity throughout the frontoparietal network (Figure S6). Next we performed the opposite manipulation and restricted short-term synaptic plasticity to presynaptic neurons in the frontoparietal network. Pinging that system did not lead to activation of the frontoparietal network (Figure S6). This suggests that synaptic plasticity at connections from (presynaptic) prefrontal cortical neurons is not required for activity-silent memory. Finally, we restricted short-term plasticity to local connections. In that network, activity-silent memory recall also failed (Figure S6). This suggests that short-term facilitation in inter-areal feedforward connections from early sensory areas to the frontal and parietal cortex is a potential substrate for “activity-silent” memory in the absence of a strong initial prefrontal response to the stimulus.

Why does the brain have two parallel systems for holding items in short-term memory? To explore this question, we simulated the model using a ping protocol (Wolff et al., 2017) with a distractor. After a behaviorally relevant cue and during the delay period, we introduced a distractor that should be filtered out by the network, followed by a neutral ping stimulus (Figure 4B, i). For mid-level dopamine release, persistent activity coding for the target stimulus is engaged and maintained through the distractor and ping (Figure 4B, ii). The distractor is represented transiently in inferior temporal (IT) and lateral intraparietal cortex (LIP) (thus replicating the experimental results in Suzuki and Gottlieb, 2013) but does not reach most of the frontoparietal network. In the low- and high-dopamine cases, during the ping, the activity-silent mechanism regenerates activity related to the last encoded stimulus, the distractor, in the frontal and parietal cortex (Figure 4B, iii). Thus, pinging from the activity-silent state scenario always recalls the latest item but cannot ignore a distractor. Therefore, dopamine release may serve to encode salient items in working memory and protect them from distraction.

### Dopamine increases distractor resistance by shifting the subcellular target of inhibition

**Figure 5.**
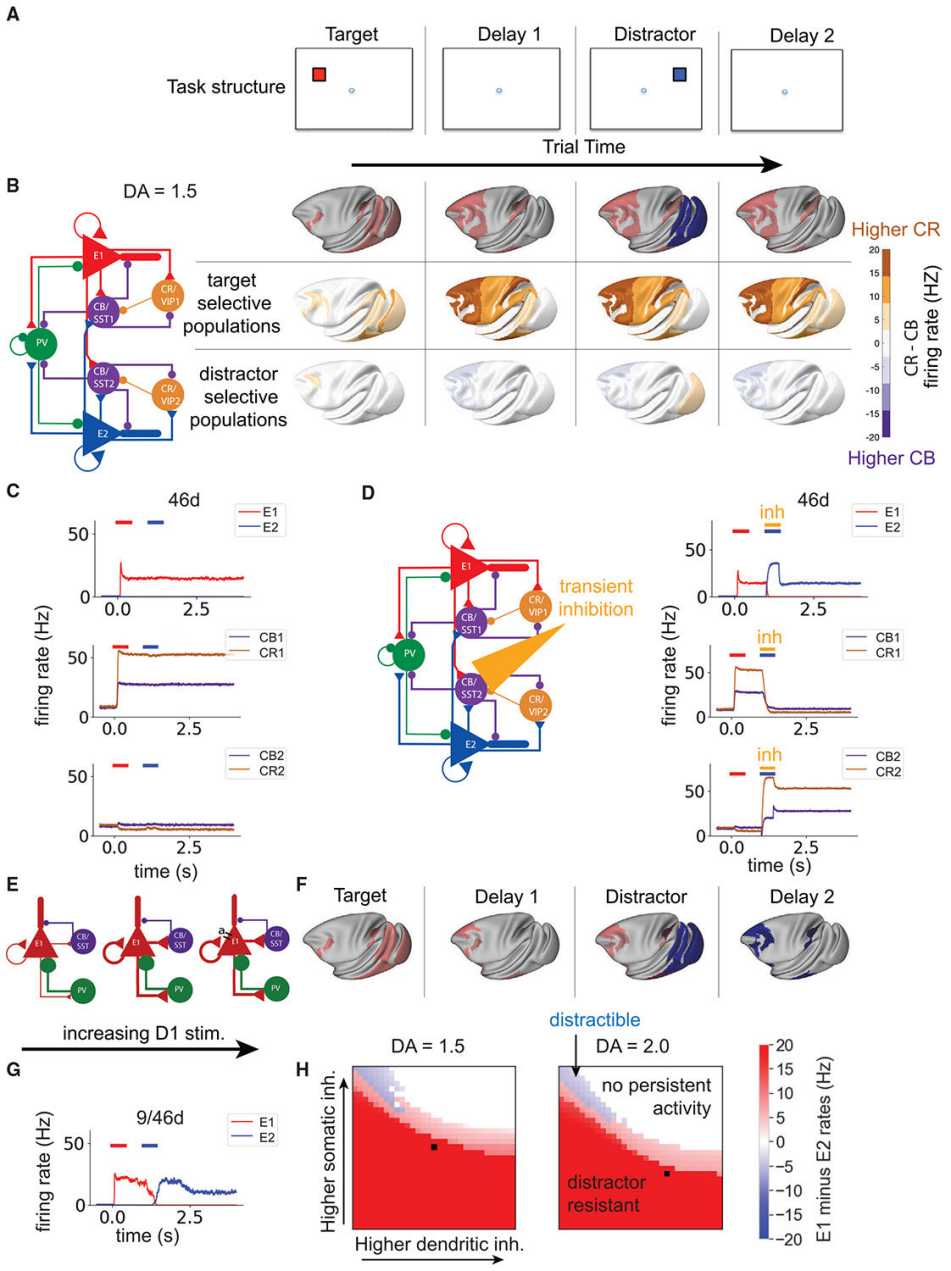
Dopamine increases distractor resistance by shifting the subcellular target of inhibition (A) Task structure. A target stimulus was followed by a delay, a distractor stimulus, and another delay period. (B) For mid-level dopamine release, persistent target-related activity (red) was present in the frontoparietal network through the delay and the distractor until the end of the trial. Each cortical area contains populations of excitatory, CB/SST, and CR/VIP cells that respond to the target stimulus (E1, CB/SST1, and CR/VIP1), separate populations sensitive to the distractor stimulus (E2, CB/SST2, and CR/VIP2), and PV cells. (B and C) Throughout the delay period and distractor stimulus, activity in CR/VIP1 is higher than in CB/SST1, leading to disinhibition of the E1 dendrite. In contrast, activity in CR/VIP2 is slightly lower than in CB/SST2, leading to inhibition of the E2 dendrite. (D) We transiently inactivated CB/SST2 populations in the frontoparietal network during presentation of the distractor stimulus. On trials in which CB/SST2 populations were inhibited, the network became distractible. (E) We removed the dopamine modulation of somatic and dendritic inhibition while leaving the effects of dopamine on NMDA-dependent excitation and adaptation unchanged. (F and G) Without the dopamine-dependent switch toward dendritic inhibition, the network became distractible, with distractor-related activity dominating at the end of the trial. (H) Consistently across dopamine levels, higher somatic and lower dendritic inhibition were associated with distractible working memory (blue). In contrast, lower somatic and higher somatic inhibition were associated with distractor-resistant working memory (red). High dendritic and high somatic inhibition result in no persistent activity (white). The levels of dendritic and somatic inhibition associated with the standard dopamine modulation used in the rest of the paper are marked by a black square. See also Figure S7.

How does dopamine protect working memory from distraction? To examine this question, we analyzed activity within CR/VIP and CB/SST neurons during a working memory task with a distractor (Figure 5A). CB/SST and CR/VIP neurons are in competition because they mutually inhibit each other. When CB/SST cell firing is higher, pyramidal cell dendrites are relatively inhibited. Conversely, when CR/VIP cell firing is higher, pyramidal cell dendrites are disinhibited. Each cortical area in the model contains two selective populations of pyramidal, CB/SST, and CR/VIP cells. We first analyzed trials in which the model successfully ignores the distractor. In the target-selective populations, CR/VIP neurons fire at a much higher rate than CB/SST neurons (Figures 5B and 5C). Thus, the dendrites of the target-selective pyramidal cells are disinhibited, allowing inter-areal target-related activity to flow between cortical areas. In the distractor-selective populations, throughout the frontoparietal network, CB/SST neurons fire at a slightly higher rate than CR/VIP cells. Thus, activity from other cortical areas is blocked from entering the dendrites of distractor-selective pyramidal cells in the frontal and parietal cortex.

To test the importance of this effect, we transiently inhibited CB/SST2 cells in the frontoparietal network during presentation of the distractor (CB/SST2; Figure 5D). This transient inhibition of CB/SST2 cells was sufficient to switch the network to a distractible state, with the distractor stimulus held in working memory until the end of the trial (Figure 5D).

Because dopamine increases the strength of inhibition to dendrites and decreases inhibition to somata, it is possible that this aspect of dopamine modulation enhances distractor resistance of the system. We removed this effect of dopamine modulation while leaving dopamine’s effects on NMDA and adaptation currents as before (Figure 5E). We repeated the working memory task in the presence of the distractor with a mid-level of dopamine, which normally results in distractor-resistant working memory. Without the dopamine-dependent shift of inhibition from the soma to the dendrite, the system becomes distractible (Figures 5F and 5G). Previous modeling work has shown that persistent activity can depend on local recurrent excitatory connections or a combination of local and inter-areal loops (Mejias and Wang, 2021; Murray et al., 2017). We searched the parameter space for the strength of local and inter-areal excitatory-to-excitatory connections and found that, when a subset of local cortical areas was endowed with sufficient recurrent excitation to generate persistent activity in isolation (e.g., $g_{E,E}^{self}$ = 0.33*nA*, *μ_E,E_* = 1.25), high somatic inhibition and low dendritic inhibition were generally associated with distractibility (Figure 5H; Figure S7). Low somatic and high dendritic inhibition were associated with distractor-resistant behavior (Figure 5H; Figure S7). Therefore, the action of dopamine in shifting inhibition from the soma to the dendrite (Gao et al., 2003), via its strong effect on CB/SST cells (Mueller et al., 2020), prevents distractor-related activity from sensory areas disrupting ongoing persistent activity in the frontoparietal network.

### Learning to optimally time dopamine release through reinforcement

In real life, we experience a constant flow of sensory inputs, and our working memory system must be flexible in determining the timing of relevant versus irrelevant information. Dopamine neurons fire in response to task-relevant stimuli (Schultz et al., 1993) but should not fire in response to task-irrelevant distracting stimuli, regardless of timing. We hypothesized that correct timing of dopamine release could be learned by simple reward-learning mechanisms.

**Figure 6.**
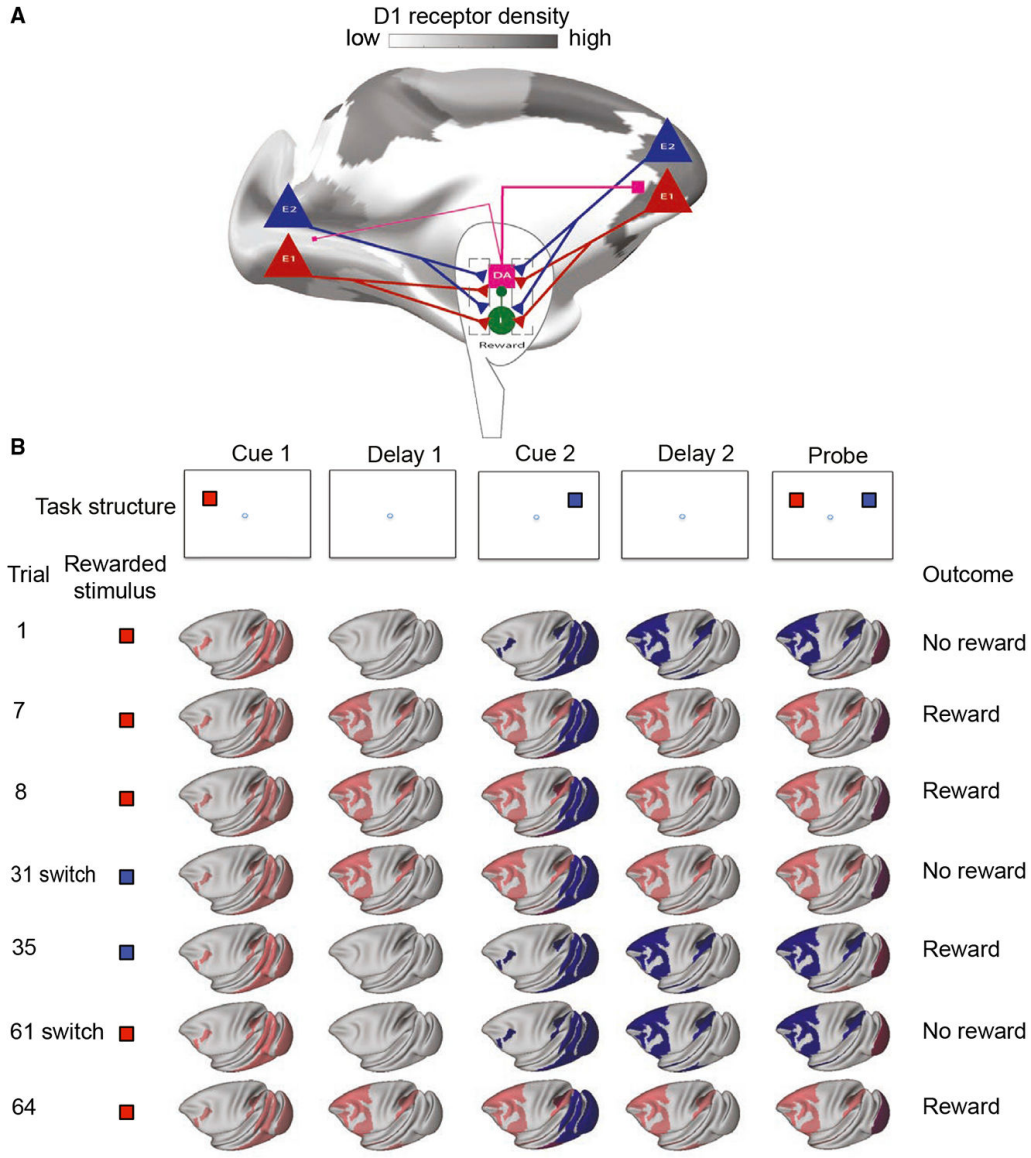
Reward-dependent learning of dopamine release appropriately engages persistent activity mechanisms to enable reversal learning (A) We designed a simplified VTA model and connected this bidirectionally to the large-scale cortical model. The VTA contained dopaminergic and GABAergic neuron populations. Dopamine was released dynamically depending on dopaminergic neuron activity. The strength of cortical inputs to VTA dopaminergic and GABAergic cells was updated at the end of each trial on the basis of trial outcome and choice. (B) We simulated a task with two cues (red and blue) followed by a probe stimulus. The rewarded stimulus changed every 30 trials. Following each switch, after a few trials, the network learns to store the appropriate stimulus in distributed persistent activity. This depends on high dopamine release in response to the rewarded stimulus and low release in response to the unrewarded stimulus.

We created a simplified model of the ventral tegmental area (VTA) with GABAergic and dopaminergic neurons and connected this to our large-scale cortical model (Figure 6A) (cf. Braver and Cohen, 2000). Cortical pyramidal cells target GABAergic and dopaminergic cells in the VTA (Soden et al., 2020;Watabe-Uchida et al., 2012). Dopaminergic cells are also strongly inhibited by local VTA GABAergic cells (Soden et al., 2020). Dopamine in the model is released in the cortex in response to VTA dopaminergic neuron firing, and cortical dopamine levels slowly return to baseline following cessation of dopaminergic neuron firing (Muller et al., 2014). In the model, the synaptic strengths of cortical inputs from the selected populations to VTA populations are increased following a reward and weakened following an incorrect response (Harnett et al., 2009; Soltani and Wang, 2006).

We tested the model on a variant of the target-distractor-ping task introduced earlier (Figures 4B, i and 6B). For the first 30 trials, the first stimulus (cue 1, red) was rewarded (rule 1). For the following 30 trials, the second stimulus (cue 2, blue) was rewarded (rule 2). For the final 30 trials, we switched back to rule 1 (Figure 6B). By the seventh trial of the first block, distractor-resistant persistent activity emerged, and the first cue was remembered correctly. This behavior persisted until the next block. Following a few trials of the second block, dopamine release in response to the first stimulus was reduced, and neural populations throughout the cortex only transiently represented the first (now irrelevant) stimulus. However, dopamine response to the second stimulus increased so that persistent activity representing the second stimulus was engaged. Following the second rule switch, the system again switched back to engaging persistent activity in response to the first cue. Additionally, the number of trials to engage appropriate persistent activity decreased gradually with each switch. We further tested the model on a version of the task in which the relevant red cue could be shown first or second within a block before the blue cue became relevant in the second block. The model was also able to learn this task, although it took more trials (10–15) to learn the switch (for the first few blocks). Thus, by means of simple reward-learning mechanisms, the optimal timing of dopamine release can be learned, allowing flexible engagement of distributed persistent activity in working memory.

## DISCUSSION

We uncovered a macroscopic gradient of dopamine D1 receptor density along the cortical hierarchy. By building a novel anatomically constrained model of the monkey cortex, we showed how dopamine can engage distributed persistent activity mechanisms and protect memories of behaviorally relevant stimuli from distraction. This work leads to new predictions that would not have been possible with local circuit models. For example, the model shows that dopamine’s enhancement of inhibition from CB/SST-expressing cells to the dendrites of pyramidal cells blocks distracting sensory information from entering the frontoparietal working memory network. Second, when an initial stimulus fails to robustly activate the prefrontal cortex, we found that the memory of the original stimulus can be recalled through an activity-silent synaptic mechanism in inter-areal connections from the sensory to the frontoparietal cortex. Last, our model predicts that dopamine can switch between activity-silent and distributed persistent activity mechanisms, and the timing of dopamine release could be learned through reinforcement. This suggests that distributed persistent activity may be engaged for behaviorally relevant stimuli that need to be remembered and protected from distractors.

### A gradient of D1 receptors along the cortical hierarchy

We used quantitative *in vitro* receptor autoradiography to create a high-resolution, high-fidelity map of cortical dopamine receptor architecture. The dopamine system can also be imaged *in vivo* using positron emission tomography (PET) and single photon emission computed tomography (SPECT) scans. These scans can provide information regarding individual and group differences but are limited in spatial resolution and signal-to-noise ratio (Abi-Dargham et al., 2002; Froudist-Walsh et al., 2017a; Roffman et al., 2016; Slifstein et al., 2015) and are often unreliable for cortical measurements (Egerton et al., 2010; Farde et al., 1988). It is now possible to map the expression of genes coding for dopamine receptors across the brain. Gene expression methods have certain advantages, especially RNA sequencing, which can provide cell-specific data. However, mRNA expression is not always closely related to or even positively correlated with the receptor density at the cell membrane (Arnatkeviciute et al., 2019; Beliveau et al., 2017). Receptor density at the membrane is the functionally important quantity and is measured here directly. The map of D1 receptor density here greatly expands previous descriptions of D1 receptor densities (Goldman-Rakic et al., 1990; Impieri et al., 2019; Lidow et al., 1991; Niu et al., 2020; Richfield et al., 1989). We show that D1 receptor density increases along the cortical hierarchy, peaking in the prefrontal and posterior parietal cortex. A previous study of 12 cortical areas suggested a posterior-anterior gradient of D1 receptor expression (Lidow et al., 1991). Here we assess D1 receptor density in 109 cortical areas, take into account variation in neuron density across the cortex, and show that the D1 receptor gradient more closely follows the cortical hierarchy than a strict posterior-anterior gradient. The distinction is clear, with higher levels of D1 receptor density per neuron in areas of the posterior parietal cortex than the somatosensory and primary motor cortex. Future work is required to test the degree to which gradients of gene expression accurately capture the receptor gradient (Beliveau et al., 2017; Hurd et al., 2001). The gradient of dopamine D1 receptors is similar to gradients of other anatomical and functional properties described across the cortex, many of which increase or decrease along the hierarchy (Burt et al., 2018; Fulcher et al., 2019; Goulas et al., 2018; Margulies et al., 2016; Sanides 1962; Shafiei et al., 2020; Wang 2020). We observed some interesting patterns of D1R density per neuron (Figure 1F), such as a gradual caudorostral increase within the prefrontal cortex, which resembles previously reported gradients of plasticity, laminar connectivity, and abstraction (Badre and D’Esposito 2009; Riley et al., 2018; Vezoli et al., 2021). Because of the small number of animals and relatively similar D1R expression levels in several areas of the frontal and parietal cortex, comparison of D1R density between pairs of areas is difficult. As shown originally in Markov et al. (2014a), the hierarchy itself is steep for early sensory areas and becomes shallower for higher-association areas. Therefore, the exact positions of areas like LIP or 10 are not as robustly distinguishable as those of V1, V2, and V4. Nonetheless, we expect the general pattern of an increase in D1R density per neuron along the cortical hierarchy to hold. Although the D1R labeling per neuron as well as synaptic excitation and inhibition display a smooth gradient, quantitative variations of circuit properties can give rise to a non-smooth pattern of persistent activity along the cortical hierarchy through a phenomenon akin to bifurcations described by the theory of nonlinear dynamical systems (Mejias and Wang, 2021; Wang, 2020). Such a sudden transition was observed in a monkey experiment where elevated persistent activity associated with working memory was absent in the middle temporal area (MT) but significantly present one synapse away in the nearby medial superior temporal area (MST) (Mendoza-Halliday et al., 2014). Simultaneous recording from many parcellated areas using new tools, such as Neuropixels (Jun et al., 2017), from behaving animals could systematically test our model prediction in future experiments. This increasing gradient of dopamine receptors along the cortical hierarchy is a major anatomical basis by which dopamine can modulate higher cognitive processing.

### An inverted U relationship between dopamine and distributed working memory activity

Previous experimental and modeling studies have shown an inverted U relationship between D1 receptor stimulation and persistent activity in the prefrontal cortex in monkeys performing working memory tasks (Brunel and Wang, 2001; Vijayraghavan et al., 2007; Wang et al., 2019). Dopamine activity in the VTA is relatively low during the delay period but still has an inverted U shape relationship with short-term memory performance in the rat (Choi et al., 2020). In our model, this may be interpreted as the VTA continuing to provide low-level dopamine to the cortex to maintain cortical dopamine levels within the appropriate bounds for distributed persistent activity. We found dense D1 and D2 receptor labeling in the striatum. However, we focused our working memory modeling on the cortex and VTA. Notably, optogenetic manipulation of *substantia nigra pars compacta* dopamine neurons (which principally target the striatum) does not have specific short-term memory effects (Choi et al., 2020). This suggests that cortical rather than striatal dopamine release is likely to be more important to short-term memory. By constructing a novel large-scale model based on the D1 receptor map and tract-tracing data, we found that the inverted U relationship between D1 receptor stimulation and persistent activity held across the frontal and parietal cortex during working memory. The working memory activity pattern was strikingly similar to that seen experimentally, according to a meta-analysis of 90 electrophysiology studies of delay period activity in the monkey cortex (Leavitt et al., 2017). Analyzing the model showed that the pattern of inter-areal connections was the strongest determinant of the pattern of working memory activity.

Noudoost and Moore (2011) found that injecting a D1 antagonist into FEF led to an increase in firing rates in V4. Similarly, in our model, when cortical dopamine levels are close to the optimal range for working memory (i.e., the peak of the inverted U), then reducing D1 receptor stimulation via an antagonist would lead to an increase in V4 activity during the second peak of the response to visual stimulation (Figure S3). However, our model focused on distributed working memory in a large-scale cortical system and was not built to uncover mechanisms of attention or decision-making. Recent electrophysiology and modeling studies of non-human primate attention have suggested that the dominant net effect of attention on neural activity in the sensory cortex is inhibition (Huang et al., 2019; Yoo et al., 2021). This may be consistent with subtle enhancement of firing for neurons whose receptive field is in the focus of attention, combined with greater inhibition of neurons with nearby receptive fields. We showed that somatosensory and visuospatial working memory tasks engage largely overlapping higher cortical areas during the delay period. It is likely that, at a neural level, these networks may overlap only partially. To simulate these mixed inhibitory and excitatory effects of attention and identify the degree to which different types of working memory engage the same neurons, future models will require more neural populations per area, perhaps with structured connectivity, such as a ring (Ardid et al., 2007). Local circuit modeling has shown previously that a circuit designed for working memory is suitable for decision-making (Wang 2002). Our model may also be suitable for considering decision processes distributed across cortical areas.

### Prefrontal and parietal contributions to distributed working memory

It is increasingly feasible to uncover the circuit mechanisms underlying distributed cognitive functions because of advances in recording technology (Jun et al., 2017) and large-scale cortical models (Cabral et al., 2011; Chaudhuri et al., 2015; Honey et al., 2007; Joglekar et al., 2018; Mejias et al., 2016; Mejias and Wang, 2021; Schmidt et al., 2018; Shine et al., 2018). Most previous large-scale cortical models have focused on replicating resting-state functional connectivity (Cabral et al., 2011; Chaudhuri et al., 2015; Honey et al., 2007) or propagation of neural activity along the hierarchy (Chaudhuri et al., 2015; Joglekar et al., 2018; Schmidt et al., 2018), with the notable exception of one recent model that simulated distributed working memory in a network of 30 cortical areas (Mejias and Wang, 2021). Compared with previous efforts, our model additionally includes (1) a D1 receptor gradient; (2) multiple inhibitory cell types and distinct pyramidal cell compartments; (3) at least 33% more cortical areas connected via quantitative graded and directed connectivity data, and, for some figures, (4) short-term synaptic plasticity; and (5) a VTA module with reinforcement learning mechanisms. The large-scale nature of the model enabled us to investigate the contributions of different brain regions to distributed working memory activity.

Some experimental studies have aimed to dissociate the contribution of the prefrontal and parietal cortex to working memory via temporary inactivations. For example, Chafee and Goldman-Rakic (2000) examined the effects of reversibly cooling the prefrontal or parietal cortex on activity in the other area and behavior during a visuospatial working memory task without a distractor. Cooling affected the FEF (area 8) and nearby prefrontal cortex, including the principal sulcus (areas 46 and 9). Cooling of the parietal cortex included LIP as well as parts of areas DP (dorsal prelunate gyrus), 7A, and 5. Cooling the parietal cortex led to a substantial reduction in prefrontal firing rates with only a minor effect on performance. Cooling the prefrontal cortex led to a substantial reduction in parietal firing rates and a large increase in behavioral errors (Chafee and Goldman-Rakic 2000). This is consistent with our simulation results showing that prefrontal and parietal inactivation can have a robust effect on reducing mnemonic delay activity but that prefrontal inactivation has much larger effects on performance (Figures 3E and 3F).

Suzuki and Gottlieb (2013) inactivated areas LIP and dorsolateral prefrontal cortex (dlPFC) using the GABA-A receptor agonist muscimol and assessed performance on a similar visuospatial working memory task with and without distractor stimuli. In these experiments, neither LIP nor dlPFC inactivation caused errors in trials without distractors (Suzuki and Gottlieb, 2013). However, inactivation of dlPFC, but not LIP, led to a dramatic increase in errors on trials with distractors (Suzuki and Gottlieb, 2013). This is consistent with our simulation results showing that precise lesions to dlPFC affect behavior on challenging working memory trials with distractor stimuli, but larger lesions are required to disrupt performance in simple working memory trials without distractors, and lesions to LIP have only subtle effects on performance. This agrees with recent models of distributed working memory that suggest that the prefrontal cortex may have a particularly important role in maintaining distributed persistent activity (Mejias and Wang, 2021; Murray et al., 2017). The effects of lesions on model performance are consistent with recent reports showing that there is a distinction between areas that are active during normal behavior and those that are essential for a computation (Pinto et al., 2019; Zatka-Haas et al., 2021) and that cortical lesions have greater effects on performance in more challenging tasks (Pinto et al., 2019).

### Lesions to areas with a high D1 receptor density disrupt working memory

Working memory activity was most disrupted by lesions to areas with a high D1 receptor density, a prediction that can be tested experimentally. Humans with traumatic brain injury often have working memory deficits (Dunning et al., 2016). Pharmacological treatment of these deficits, including with dopaminergic drugs, has had mixed success (Froudist-Walsh et al., 2017b). Our model simulations suggest that D1 agonists or antagonists could be effective at restoring normal working memory functioning following lesions to particular cortical areas, but the correct treatment may depend on the baseline cortical dopamine levels of the individual. Dopaminergic drugs have also been suggested as treatments for individuals with schizophrenia with working memory deficits (Yang and Chen 2005). In individuals with schizophrenia, PV and SST gene expression is reduced across multiple areas of the cortical working memory network (Tsubomoto et al., 2019). Disruption of these inhibitory neurons is likely to contribute to working memory deficits. Future adaptations of our model could allow simulation of working memory deficits and motivate potential treatments for individuals based on their particular anatomy, gene expression, and patterns of cortical dopamine release or receptor density (Abi-Dargham et al., 2002; Slifstein et al., 2015).

### A dopamine switch between the activity-silent state and persistent activity

For very low or high levels of D1 receptor stimulation, it was possible to maintain stimulus information in the absence of persistent activity via synaptic mechanisms. This pattern of successful memory recall without frontoparietal delay period activity is reminiscent of a passive short-term memory trace thought to rely on “activity-silent” synaptic mechanisms (Rose et al., 2016; Trübutschek et al., 2017; Wolff et al., 2017) that could occur without ignition of the frontoparietal cortex (Trübutschek et al., 2017, 2019). Previous models with short-term synaptic plasticity have focused on local activity in the prefrontal cortex (Mongillo et al., 2008) and, thus, implicitly imply that the initial stimulus must significantly engage prefrontal neural activity and store the memory trace via short-term plasticity in local prefrontal connections. However, some stimuli may be remembered without a strong initial prefrontal response. We found that short-term synaptic plasticity in inter-areal connections from sensory to frontoparietal areas was most important for maintaining the silent memory trace. In particular, this is a potential mechanism for activity-silent short-term memory in the absence of a strong initial prefrontal response to the stimulus. It has been proposed that nonspecific excitatory or inhibitory currents could account for switches between active and silent states (Barbosa et al., 2020). Our model suggests that dopamine could, in fact, account for the switch from the silent to the active state. Indeed, because of the inverted U relationship between dopamine and persistent firing, a dopamine response to the reward at the end of a trial could also terminate persistent activity. Another recent proposal suggests that activity-silent short-term memory could be undertaken via hippocampal-prefrontal episodic memory mechanisms, perhaps in combination with short-term synaptic changes in the cortex (Beukers et al., 2021). Future studies should aim to disentangle the contributions of rapid synaptic changes within the prefrontal cortex (Mongillo et al., 2008), at inter-areal connections from sensory areas (this paper), or in the hippocampus (Beukers et al., 2021) to activity-silent short-term memory in the primate. We found that, in the activity-silent state, the most recently encoded stimulus was always encoded most strongly, even when it was a distractor. This may reflect involuntary encoding of irrelevant stimuli in a short-term synaptic memory trace (Barbosa et al., 2021,^2020^). This prediction should hold as the number of distractors is increased. The activity-silent system may still be able to recall earlier stimuli for a limited time when another input biases the network toward the activity pattern used during encoding of the earlier stimulus to trigger pattern completion and recall of the memory (Manohar et al., 2019) or through active forgetting of the distracting stimuli (Wolff et al., 2021). Alternatively, multiple competing memories may be represented in neural activity (Barbosa et al., 2021; Panichello and Buschman, 2021), which would rely on an unspecified selection mechanism and may occur in parallel with short-term synaptic changes. In our model, stimuli stored in persistent activity (and thus dependent on mid-level dopamine release) were more robust against distraction, consistent with drug studies in humans (Fallon et al., 2017a, 2017b). Thus, dopamine release may engage distributed persistent activity to protect memories of important stimuli from distraction.

### Dopamine increases distractor resistance by shifting the subcellular target of inhibition

The resilience of the active working memory state in the model depended on CB/SST cells blocking distracting inputs from sensory areas to the dendrites of pyramidal cells in the frontal and parietal cortex. Previous modeling work on local cortical circuits has suggested that greater dendritic and less somatic inhibition could increase distractor resistance (Wang et al., 2004a) and that selective disinhibition of the dendrite (through CR/VIP cells) could allow specific information to be passed through the network (Yang et al., 2016). In our large-scale model, CR/VIP cells selectively disinhibited the dendrites of target-selective cells, allowing target-related activity to flow through the cortical network. D1 receptors in the monkey cortex are more strongly expressed on CB/SST neurons than other interneuron types (Mueller et al., 2020). In agreement with these anatomical findings, application of dopamine to a frontal cortex slice increases inhibition to the dendrites and decreases inhibition to the somata of pyramidal cells (Gao et al., 2003). We found that, as long as local cortical areas (or potentially cortico-subcortical loops) are capable of maintaining persistent activity, then shifting the balance of inhibition from the soma to the dendrite can allow maintenance of an active representation of a stimulus in persistent activity while shielding it from distracting input from sensory areas. The ability of cortical areas to maintain persistent activity itself depends on dopaminergic enhancement of NMDA-dependent excitation. In mice, inhibition of SST neurons in medial prefrontal cortex during the sample period of a spatial working memory task impairs performance and increases representation of irrelevant information in prefrontal activity (Abbas et al., 2018). Consistent with our model, this suggests that SST neurons gate entry of information into working memory and that inhibition of SST neurons in frontoparietal areas allows distracting information to enter.

### Learning to engage distributed persistent activity through reinforcement

Distractor resistance in response to all stimuli could render the working memory system inflexible and unresponsive to new, potentially important inputs. Previous studies have shown that lesioning the prefrontal cortex impairs the ability to switch attention between stimuli across trials (Rossi et al., 2007). Our model predicts that the prefrontal cortex is more crucial for persistent activity than activity-silent short-term memory, which can rely on short-term synaptic changes outside of the prefrontal cortex. We show that, by using a simple reward-based learning mechanism, a cortical VTA model (cf. Braver and Cohen, 2000; Frank 2005) can successfully perform a task with reversals between the memory cue and distractor stimuli across trials. In our model, the timing of dopamine release in the cortex can be learned to engage distributed persistent activity throughout the frontoparietal network only in response to reward-predicting cues. Dopamine neurons burst about 130–150 ms after reward-predicting stimuli, coinciding with a rise in activity in frontal cortical neurons (de Lafuente and Romo, 2012). Because of the slow dynamics of cortical dopamine (Muller et al., 2014), we suggest that a transient increase in dopamine release in response to the target stimulus (Choi et al., 2020; Schultz et al., 1993) may be sufficient to maintain distributed persistent activity for several seconds. This mechanism may thus be reserved for behaviorally important stimuli that must be protected from distraction even when the behaviorally relevant stimuli change from trial to trial. In contrast, irrelevant or less salient stimuli result in lower dopamine release and may be remembered via silent mechanisms or forgotten. We investigated model performance on a reversal learning task with identical repeated trials within a block. In natural life, no two situations are exactly the same. It is likely that the brain generalizes across similar situations to enable reinforcement learning to be used in practice. This ability to generalize may arise from dopamine-dependent plasticity in the prefrontal cortex (Wang et al., 2018). The classic reward-prediction-error hypothesis treats dopamine as a global scalar reward prediction error signal that is spatiotemporally uniform (Schultz 1998). Here we aim to highlight one form of spatial heterogeneity and suggest that broad dopamine release will affect each cortical area according to the D1 receptor density per neuron. Recent work suggests that there is temporal heterogeneity in dopamine release, which is released in waves in the mouse striatum (Hamid et al., 2021). Whether such dopamine waves also occur in the cortex or in primates remains to be seen. Even if dopamine is released in waves across the cortex, its effect on cortical areas will be dependent on the D1 receptor gradient presented here.

### Roles of other neuromodulatory and subcortical systems

In addition to dopamine, other neuromodulators, such as acetylcholine (Croxson et al., 2011; Sun et al., 2017; Yang et al., 2013) and noradrenaline (Arnsten et al., 2012), affect prefrontal delay period firing and performance on visuospatial working memory tasks. Cholinergic mechanisms may complement dopaminergic mechanisms. For example, nicotinic alpha-7 receptors depolarize pyramidal cells, which enables NMDA receptors to be engaged via removal of the magnesium block (Yang et al., 2013). This may compensate for a reduction in presynaptic glutamate release in response to D1 stimulation and enable dopamine’s permissive effects on NMDA transmission (Seamans et al., 2001). Muscarinic M1 receptor activation closes KCNQ channels, which contribute to the hyperpolarizing effect of high levels of D1 stimulation (Arnsten et al., 2012; Galvin et al., 2020). Thus M1 stimulation may enable persistent activity over a larger range of dopamine release. The effects of noradrenaline on working memory circuits depend on the targeted adrenergic receptors. Moderate release of noradrenaline engages adrenergic *α*_2*A*_ receptors, which may counteract the hyperpolarizing effects of hyperpolarization-activated cyclic nucleotide-gated (HCN) channels (Arnsten, 2000; Arnsten et al., 2012; Li and Mei, 1994; Robbins and Arnsten, 2009) and keep the D1 effects in check by decreasing calcium-cyclic AMP (cAMP) signaling. Greater noradrenergic levels engage a1 and b1 receptors, which promote calcium-cAMP signaling and, at high levels, provide negative feedback via KCNQ and HCN channels (Arnsten et al., 2020). Studies linking neuromodulators to working memory have focused on the dorsolateral prefrontal cortex. Much less is known about the influence of these and other neuromodulators on the distributed network activity that underlies working memory outside of the prefrontal cortex. Future work should focus on the interaction of distinct neuromodulators and how release of different combinations of neuromodulators may affect distributed activity patterns and behavior, taking into account the different distributions of these receptors across the cortex (Froudist-Walsh et al., 2021). Subcortical structures, such as the thalamus, may play a significant role in working memory (Fuster and Alexander, 1971; Guo et al., 2017; Jaramillo, et al., 2019; Watanabe and Funahashi, 2012). Future experiments and computational modeling studies should aim to disentangle the contribution of the thalamus to sensory working memory and motor preparation (Guo et al., 2017; Watanabe and Funahashi, 2012) and clarify the degree to which such mechanisms are shared across species. When appropriate weighted and directed connectivity data become available, future large-scale cortical models should also integrate further structures, such as the thalamus (Jaramillo et al., 2019), basal ganglia (Wei and Wang, 2016), the claustrum, and the cerebellum to identify their contributions to working memory.

### Conclusion

We experimentally found a macroscopic gradient of dopamine D1 receptor density along the cortical hierarchy. By building a novel connectome-based biophysical model of the monkey cortex, endowed with multiple types of inhibitory cells, we show how dopamine can engage robust distributed persistent activity mechanisms across connected higher cortical areas and protect memories of salient stimuli from distraction. Because distributed persistent activity is necessary for internal manipulation of information in working memory (Masse et al., 2019; Takeda and Funahashi, 2004; Trübutschek et al., 2019), dopamine release in the cortex may be a key step toward higher cognition and thought.

## STAR★METHODS

### RESOURCE AVAILABILITY

#### Lead contact

Further information and requests for resources should be directed to and will be fulfilled by the lead contact, Xiao-Jing Wang (xjwang@nyu.edu).

#### Materials availability

This study did not generate new unique reagents.

#### Data and code availability

Dopamine D1 receptor per neuron and tract-tracing connectivity data have been deposited at at BALSA: 7qKNZ and core-nets and are publicly available as of the date of publication. Accession numbers are listed in the Key resources table.

All original code has been deposited at GitHub: seanfw/dopamine-dist-wm and Zenodo: https://doi.org/10.5281/zenodo.5507279 and is publicly available as of the date of publication. DOIs are listed in the Key resources table.

Any additional information required to reanalyze the data reported in this paper is available from the lead contact upon request.

### EXPERIMENTAL MODEL AND SUBJECT DETAILS

For *in-vitro* receptor autoradiography we analyzed the brains of three adult male *Macaca fascicularis* specimens (between 6 and 8 years old; body weight between 5.2 and 6.6 kg) obtained from Covance (now Labcorp Drug Development), Münster, where they were used as control animals for pharmaceutical studies performed in compliance with legal requirements. All experimental protocols were in accordance with the guidelines of the European laws for the care and use of animals for scientific purposes.

Tract tracing data was obtained from fluorescent retrograde injections of fast blue (FsB) and diamidino yellow (DY) in 29 areas reported in Markov et al., 2014b supplemented by injections in an additional 11 areas with either FsB (areas 9, OPRO), DY (areas LIP, V6, 25, 32) or cholera toxin subunit B (CTB) (areas 1, 3, 45A, F4, F3). Animals were aged 10-15 years, female and M. fasicularis except for the LIP injection which was M. mulatta. The LIP injection was reported in Mejias et al. (2016). Animals were group housed in cages in with access to plastic toys and other enrichment devices. Housing and surgical intervention were in accordance with European procedures and were reviewed by the veterinary and ethical services.

### METHOD DETAILS

#### Overview of anatomical data

In this study, we combine post-mortem anatomical data on receptor densities, white matter connectivity, neuron densities and dendritic spine counts. Each of these four anatomical measures was originally quantified using different parcellations of cortex. Large sections of the temporal lobe are not yet quantified for either the receptor autoradiography data, or the tract-tracing connectivity data. Collection of this data is underway and will be made available in future studies. With the exception of the receptor densities in the posterior parietal cortex (Impieri et al., 2019; Niu et al., 2020, 2021), all D1 receptor densities are reported for the first time in this study. The connectivity data for ten of the 40 cortical areas is used here for the first time, but will be described in more detail in an upcoming publication from the Kennedy lab. This enabled us to expand the calculation of the cortical hierarchy to 40 regions.

#### A note on notation

Subscripts in square brackets, such as [*k*] are used to denote cortical areas themselves. Subscripts not in brackets, such as *i* are used to denote populations of neurons within a cortical area. Superscripts are used to provide further clarifying information. We use the convention that targets are listed before sources, so that *g_i,j_* would denote the strength of a connection from neural population j to neural population i. Parameter values are listed in Table S6.

#### Quantification of receptor density across cortex - in-vitro autoradiography

In order to create a high-resolution, and high-fidelity map of cortical dopamine receptor architecture, we used quantitative *in-vitro* receptor autoradiography (Palomero-Gallagher and Zilles, 2018). Previous dopamine receptor autoradiography has focused on relatively small sections of cortex (Goldman-Rakic et al., 1990; Impieri et al., 2019; Lidow et al., 1991; Niu et al., 2020; Richfield et al., 1989). To create a more comprehensive map of the cortical dopamine receptors, we measured D1 receptor density across 109 cortical areas, and D1 and D2 receptors in the basal ganglia.

Animals were sacrificed by means of an intravenous lethal dose of sodium pentobarbital. Brains were removed immediately from the skull, and brain stem and cerebellum were dissected off in close proximity to the cerebral peduncles. Hemispheres were separated and then cut into a rostral and a caudal block by a cut in the coronal plane of sectioning between the central and arcuate sulci. These blocks were frozen in isopentane at −40C to −50C, and then stored in airtight plastic bags at −70C. Each block was serially sectioned in the coronal plane (section thickness 20 μm) using a cryostat microtome (CM 3050, Leica, Germany). Sections were thaw-mounted on gelatine-coated slides, freeze-dried overnight and processed for visualization of D1 or D2 receptors, cell bodies (Merker, 1983) or myelin (Gallyas, 1979).Quantitative *in-vitro* receptor autoradiography was applied to label dopaminergic D1 and D2 receptors according to previously published protocols (Palomero-Gallagher and Zilles, 2018) (Zilles et al., 2002) encompassing a preincubation, a main incubation and a final rinsing step. For visualization of the D1 receptor, sections were first rehydrated and endogenous substances removed during a 20 minute preincubation at room temperature in a 50 mM Tris-HCl buffer (pH 7.4) containing 120 mM NaCl, 5 mM KCl, 2 mM CaCl2 and 1 mM MgCl2. During the main incubation, sections were incubated with either 0.5 nM [3H]SCH 23390 alone (to determine total binding), or with 0.5 nM [3H]SCH 23390 and 1 mM of the displacer mianserin (to determine the proportion of displaceable, non-specific binding) for 90 minutes at room temperature in the same buffer as used for the preincubation. Finally, the rinsing procedure consisted of two 20 minutes washing steps in cold buffer followed by a short dip in distilled water. For visualization of the D2 receptor, sections were preincubated with 50 mM Tris-HCl buffer (pH 7.4) containing 150 mM NaCl and 1% ascorbate. In the main incubation, sections were incubated with either 0.3 nM [3H]raclopride alone, or with 0.3 nM [3H]raclopride and 1 μM of the displacer 1 μM butaclamol for 45 minutes at room temperature in the same buffer as used for the preincubation. Rinsing consisted of six 1 minute washing steps in cold buffer followed by a short dip in distilled water. Specific binding is the difference between total and non-specific binding. Since the ligands and binding protocols used resulted in a displaceable binding, which was less than 5% of the total binding, total binding is considered to be equivalent of specific binding. Sections were dried in a cold stream of air, exposed together with plastic scales of known radioactivity against tritium-sensitive films (Hyperfilm, Amersham) for six (for the D1 receptor) or eight (for the D2 receptor) weeks, and ensuing autoradiographs processed by densitometry with a video-based image analyzing technique (Palomero-Gallagher and Zilles, 2018)(Zilles et al., 2002). Autoradiographs were digitized using a CCD-camera, and stored as 8-bit gray value images with a spatial resolution of 2080x1542 pixels. Grey values (*g*) in the co-exposed scales as well as experimental conditions were used to create a regression curve with which gray values in each pixel of an autoradiograph were transformed into binding site densities (Bmax) in fmol/mg protein by means of the formula

(Equation 1) $$B_{max}=\frac{gR}{EBW^{b}s^{a}}\cdot \frac{K^{D}+L}{L}$$

where *R* is the radioactivity concentration (cpm) in a scale, *E* the efficiency of the scintillation counter used to determine the amount of radioactivity in the incubation buffer, *B* the number of decays per unit of time and radioactivity, *W^b^* the protein weight of a standard, *s^a^* the specific activity of the ligand, *K^D^* the dissociation constant of the ligand, and *L* the free concentration of the ligand during incubation. For visualization purposes solely, autoradiographs were subsequently pseudo-color coded by linear contrast enhancement and assignment of equally spaced density ranges to a spectral arrangement of eleven colors.

Cortical areas were identified by cytoarchitectonic analysis and receptor densities measured at comparable sites in the adjacent sections processed for receptor visualization. The mean receptor density for each area over a series of 3–5 sections per animal and receptor was determined by density profiles extracted vertical to the cortical surface using MATLAB-based in house software (Palomero-Gallagher and Zilles, 2018).

#### Retrograde tract-tracing

The inter-areal connectivity data in this paper is part of an ongoing effort to map the cortical connectome of the macaque using retrograde tract-tracing (Markov et al., 2013, 2014a, 2014b). For each target area, a retrograde tracer was injected into the cortex. The tracer was taken up in the axon terminals in this area, and retrogradely transported to the cell bodies of neurons that projected to the target. These cell bodies could be throughout the brain. Each of these cell bodies in cortex was counted as a labeled neuron (LN). The amount of labeled neurons was counted in all cortical areas except for the injected target area. The cortical areas that send axons to the target area are called source areas. As there are uncontrollable differences in tracer volume and uptake between injections, we estimated the strength of connections as follows. For a given injection, the total number of cell bodies in the cortex outside of the injected (target) area was counted. The number of labeled neurons within a source cortical area was then divided by the number of labeled neurons in the whole cortex (excluding the target area), to give a fraction of labeled neurons (FLN). The FLN was averaged across all injections in a given target area. For this calculation, we include all areas in the entire cortical hemisphere (*n^areas^* = 91).

(Equation 2) $$FLN_{\left[k,l\right]}=\frac{LN_{\left[k,l\right]}}{\sum_{l=1}^{n^{areas}}LN_{\left[k,l\right]}}$$

In addition, for each connection we defined the supragranular labeled neurons (SLN) as the fraction of neurons in the source area whose cell bodies were in the superficial (aka supragranular) layers.

(Equation 3) $$SLN_{\left[k,l\right]}=\frac{LN_{\left[k,l\right]}^{supra}}{LN_{\left[k,l\right]}^{supra}+LN_{\left[k,l\right]}^{infra}}$$

The subiculum (SUB) and piriform cortex (PIR) have a qualitatively different laminar structure to the neocortical areas, and thus supra- and infra-laminar connections (and thus the SLN) from these areas are undefined. We thus removed all connections from these areas from the following calculations (*n^areas,SLN^* = 89). These connectivity data are available on the core-nets website.

#### Estimation of the cortical hierarchy

Following (Markov et al., 2014a), we estimate the hierarchical position *h* of each area using the SLN values of its connections. Feedforward connections tend to originate in the supragranular layers, while feedback connections tend to originate in the deep layers of the source area (Barone et al., 2000; Felleman and Van Essen, 1991). Moreover, if a target area occupies a much higher hierarchical position than the source area, a greater proportion of the neurons emerge from the supragranular layers of the source area than if the two areas are closer in the hierarchy (Barone et al., 2000). Likewise for the feedback connections, a greater hierarchical distance between the areas implies that the higher area sends a greater proportion of it projections from the infragranular layers. This implies that the fraction of neurons coming from the supragranular layers in a given connection gives an estimate of the relative hierarchical position of two connected areas (Barone et al., 2000; Markov et al., 2014a). Here, following (Markov et al., 2014a), we estimate a set of hierarchical levels (one per area) that best predicts the SLN values for all connections in the dataset.

The model to estimate the hierarchy has the form

(Equation 4) g(E(SLN))=Xβ

where g is a function that links the SLN of the connection between areas to the hierarchical distance between them. β is a column vector of length *n^areas,SLN^*, containing the hierarchy values to be estimated. *X* is an incidence matrix of shape *n^conns^*×*n^areas,SLN^*, where *n^conns^* ( = 2619) is the number of observed (non-zero) connections between cortical areas in the remaining dataset. Each row in *X* represents a connection, and each column represents a cortical area. All entries in each row equal 0 except for the column corresponding to the source area, which has a value of −1, and the target (recipient) area, which has a value of 1 (Strang, 1993).

The hierarchical values can be estimated with maximum likelihood regression. However, the model is singular (the rows sum to zero). In order to make the model identifiable, we therefore removed one column from *X*. We chose to remove the column corresponding to area V1, which is therefore forced to have a hierarchical value of 0. However, the choice of column is unimportant, as it is possible to estimate negative hierarchical values (in the case that other areas are lower than V1 in the hierarchy).

We used the beta-binomial model. The binomial parameter *p* corresponds to the proportion of successes. This is thought to be a random variable following a Beta distribution. The beta-binomial distribution depends on two parameters, the mean (μ, here the SLN), and the dispersion (ϕ). The beta-binomial model can account for the overdispersion of the neural count data. Note that the SLN of each measured connection is input into the model, without averaging across repeated injections.

The likelihood is written as

(Equation 5) $$f(\mu ,\phi ;q,n)=\left(\begin{array}{c} n\\ q \end{array}\right)\frac{B\left(\mu \left(\frac{1-\phi }{\phi }\right)+q,(1-\mu )\left(\frac{1-\phi }{\phi }\right)+n-q\right)}{B\left(\mu \left(\frac{1-\phi }{\phi }\right),(1-\mu )\left(\frac{1-\phi }{\phi }\right)\right)}$$

where *q* is the number of neurons projecting from the supragranular layers, n is the number of neurons projecting from all layers, and B is the beta function defined as

(Equation 6) $$B(x,y)=\int_{0}^{1}p^{x-1}(1-p{)}^{y-1}\;dp$$

with *x,y* > 0. We fit the model using *μ* = Φ(*Xβ*), where Φ is the cumulative Gaussian, as it maps the real numbers to the (0,1) range. Φ^−1^ = *g* in Equation 4 is the probit link function. The hierarchy is estimated by minimizing the log-likelihood. For more details see Markov et al. (2014a).

We then rescaled the hierarchy so that the maximum hierarchial value within the 40 region complete subgraph (containing all injected areas) equaled 1:

(Equation 7) $$h_{\left[k\right]}=\frac{\beta_{\left[k\right]}}{max\left(\beta^{subgraph}\right)}$$

for all cortical areas *k* in the complete 40-area subgraph.

For the circular embedding of the connectivity data, we estimate angles *θ_i,j_* between areas *A_i_* and *A_j_* so that a smaller angular distance between areas corresponds to a higher connectivity strength (Chaudhuri et al., 2015). The dissimilarity *d*(*A_i_*, *A_j_*) is defined as

d(Ai,Aj)={−log10(FLN(Ai,Aj))forFLN(Ai,Aj)≥0−log10(FLNmin)forFLN(Ai,Aj)=0}

where *FLN_min_* = 10^−7^, a value smaller than any FLN in the dataset.

The angles *θ_i_* are assigned to each area such that

$$d(A_{i},A_{j})\approx min(|\theta_{i}-\theta_{j}|,2\pi -|\theta_{i}-\theta_{j}|)$$

The estimated angles *θ_i_* are constrained to lie within the range [0, 1] and then mapped onto [0, 2*π*].

The radial distance from the center of the circle is $r_{i}=\sqrt{1-h_{i}}$, where *h_i_* is the hierarchical value of the area, as defined above.

#### Integration of anatomical datasets

All anatomical data was mapped to the appropriate parcellations on the Yerkes19 surface. For the present study, we mapped all data to the 40 area Lyon subgraph (Markov et al., 2014b), as the areas in this parcellation were generally larger than those in the Julich Macaque Brain Atlas (Impieri et al., 2019; Niu et al., 2020; Rapan et al., 2021; this paper) and the Queensland (spine count) injection sites (Elston, 2007), and closer to standard areal descriptions than the Vanderbilt (neuronal density) (Collins et al., 2010) sections.

The receptor densities were quantified in 109 cortical regions defined by cyto- and receptor-architecture. The method for the delineation of cortical region borders is described in (Impieri et al., 2019; Niu et al., 2020; Rapan et al., 2021). Using the same method, anatomists (NPG, MN, LR) identified cortical areas on the basis of the receptor and cyto-architecture. See Figure 1 for the definition of the areas. Anatomists carefully drew (NPG, MN, LR) and independently revised (NPG, MN, LR, SFW) defined borders on the Yerkes19 cortical surface (Donahue et al., 2016) to enable comparison with other data types. The D1 receptor data was mapped to the Lyon atlas as follows. For each area in the Lyon atlas, we searched for overlaps with areas in the Julich Macaque Brain Atlas. If more than 50% of the vertices within the area were also in the Julich Macaque Brain Atlas, the D1 receptor density for the area was calculated. All vertices within each Julich area were assigned the mean value for that area. We averaged the D1 receptor density across all vertices that lay within both the Lyon area and the Julich Macaque Brain Atlas, thus performing a weighted average of the D1 receptor densities according to the degree of spatial overlap. Thirty-two of the 40 Lyon areas were assigned D1 receptor density in this way, with the remaining eight areas not overlapping sufficiently with the Julich Macaque Brain Atlas. Due to the strong positive correlation between the D1 receptor/neuron density and the hierarchy (Figure 1), for the simulations we inferred values for the remaining eight regions using linear regression with hierarchy as the independent variable and D1 receptor/neuron density as the dependent variable.

The *in-vitro* autoradiography data accurately quantifies the density of receptors across cortex. However, it is important to bear in mind that the density of neurons also varies across the cortex. Collins et al. (2010) measured the density of neurons across the entire macaque cortex using the isotropic fractionator (a.k.a. brain soup) method. In the original paper, the cortex was divided into 42 regions and displayed on a flatmap, with anatomical landmarks labeled (Figures 2 and S1 of that paper). The borders of these regions were drawn on the Yerkes19 surface by SFW with reference to the original paper (Collins et al., 2010), several anatomical papers from the same group (Beck and Kaas, 1999; Cerkevich, et al., 2014; Kaas, 2004), the Julich Macaque (109 areas) and the Lyon (Markov-132) atlases (Donahue et al., 2016; Markov et al., 2014b), and were independently assessed by anatomists (LR, MN, NPG). The neural density data covered the entire cortex. As such, we assigned neural density to each area in the Lyon atlas, weighted by the spatial overlap with the original regions in the Vanderbilt atlas. D1 receptor density was divided by the neuron density to give the D1 receptor/neuron density in each area. The neuron density was in units of neurons per gram. To estimate the receptor density in fmol per neuron, we used the previously reported figure that 8% of brain tissue is protein (McIlwain and Bachelard, 1972). This amounts to multiplying by a constant, and does not affect the correlations or the effect of the dopamine gradient in the model.

The Lyon atlas used to define the interareal connectivity data (Markov et al., 2014b) is already available on the Yerkes19 surface (Donahue et al., 2016). The complete subgraph of injected areas including bidirectional connectivity has been expanded from 29 areas in Donahue et al. (2016) to the 40 areas used in this paper.

For the spine count data, outlines of the 27 injection sites were drawn on the Yerkes19 surface by SFW with reference to the original papers (most of which had substantial anatomical description and hand-drawn maps), as well as anatomical papers cited within the original papers (Cavada and Goldman-Rakic, 1989; Preuss and Goldman-Rakic, 1991; Seltzer and Pandya, 1978) and the Lyon and Julich Macaque Brain Atlases. Direct comparison with the hand-drawn maps was possible for areas V1, V2, MT, LIPv, 7a, V4, TEO, STP, IT, Ant. Cing., Post. Cing, TEpd, 12vl, A1, 3b, 4, 5, 6, 7b, 9, 13, 46, 7 m (Elston, 2007). Areas 10, 11 and 12 were described with reference to Preuss and Goldman-Rakic (1991). The injection in area TEa used the maps in Seltzer and Pandya (1978) for area definition. We used these maps to approximate the injection location. Area STP was identified with the corresponding region STPp in the atlas of Felleman and Van Essen (1991). Area FEF was identified as lying on the anterior bank of the medial aspect of the arcuate sulcus, as described by Elston (2007). All identified injection sites on the cortical surface were independently verified by MN, LR and NPG. Spine count data was expressed according to injection sites, rather than entire cortical areas. As such, we found the number of vertices from each injection site overlapping with each area in the Lyon atlas. For each Lyon area, the spine count was an average of the spine counts for all the injection sites overlapping with the area, weighted by the number of vertices of each injection site contained within the area. In this way we estimated the spine counts on pyramidal cells in 24 of the 40 regions in the Lyon atlas. Based on the strong positive correlation between spine count and cortical hierarchy (r = 0.61, p = 0.001), and following previous work (Chaudhuri et al., 2015; Mejias and Wang 2021), we inferred the spine count for the remaining regions based on the hierarchy using linear regression.

Neuroanatomists (NPG, LR, MN) classified each of the 109 cortical areas for which D1 receptor data is available as being either granular, or agranular, and according to the ratio of cell body size between layers III and V.

Delineations of the areal borders for each atlas, and the anatomical data in the Yerkes19 space are available on the BALSA database.

#### Overview of dynamical models

We first describe the connectivity structure of our local circuit model, and how dopamine modulates the efficacy of these connections. We then describe a large-scale dynamical model, in which the local circuit is used as a building block, and placed in each of 40 cortical areas. We describe the various steps to building the large-scale model, including how to connect the cortical areas, apply heterogeneity of excitation and the gradient of dopamine. Lastly, we describe how we simulated working memory tasks, lesions and transient inhibition in this model.

#### Description of the local cortical circuit

We describe a local cortical circuit containing populations of four distinct types of neurons. This is conceptually related to previous computational models of working memory involving multiple types of interneurons (Tanaka, 1999; Wang et al., 2004a), and uses a mean field reduction of a spiking model (Brunel and Wang, 2001; Wong and Wang, 2006). PV, CB/SST and CR/VIP cells differed in the threshold and slope of their input-output function (f-I curve) (Bacci et al., 2003), local (Adesnik et al., 2012; Jiang et al., 2015; Muñoz et al., 2017; Pfeffer et al., 2013; Tremblay et al., 2016) and long-range connectivity (Lee et al., 2013; Wall et al., 2016), adaptation rates (Kawaguchi, 1993; Mendonça et al., 2016; Schuman et al., 2019), and NMDA/AMPA ratio (Lu et al., 2007).

The connectivity structure and strengths of the local circuit, are based on a synthesis of anatomical and physiological studies, and are captured in the local connectivity matrix *G* (Tables S1-S3; Jiang et al., 2015; Kalisman et al., 2005; Lee et al., 2013; Ma et al., 2012; Markram et al., 1997; Pfeffer et al., 2013; Silberberg and Markram, 2007; Walker et al., 2016). Note that connection probability and synaptic strength between neural types are generally positive correlated (Jiang et al., 2015). This simplifies the process of identifying the relative strengths of connections between neural populations in the circuit.

We grouped the pyramidal neurons into two separate populations. Each of these populations is selective to a particular visual feature (such as a region of visual space). Pyramidal cells excite all cell types in the circuit, with different strengths. We model two compartments in the pyramidal cells. One compartment represents the soma and proximal dendrites, and the other the distal dendrites. The dendrite is modeled as a simplified nonlinear function, adapted from Yang et al. (2016). Pyramidal cells target the soma and proximal dendrites of other pyramidal cells in the same cortical area (Kalisman et al., 2005; Markram et al., 1997; Petreanu et al., 2009). Each type of inhibitory neuron has a unique pattern of connectivity. The first inhibitory cell type targets the perisomatic area of the pyramidal cells. These cells express parvalbumin (PV) and are fast spiking (Jiang et al., 2015; Kawaguchi, 1993,^1995^). They are basket cells with axons that branch across wide distances, which allows them to inhibit pyramidal cells in neighboring populations (Helmstaedter et al., 2009; Kawaguchi, 1995). They also inhibit other PV neurons (Jiang et al., 2015; Pfeffer et al., 2013). Compared to other inhibitory neurons, PV neurons receive a smaller proportion of excitatory inputs via NMDA receptors (Lu et al., 2007; Wang and Gao, 2009). The second type of inhibitory neuron targets the distal dendrites of excitatory cells. In non-human primates, dendrite-targeting cells express calbindin (DeFelipe et al., 1989). The best characterized dendrite-targeting cell type in rodents is the Martinotti cell, which expresses somatostatin (CB/SST) (Wang et al., 2004b). These cells target all other cell types, while avoiding other Martinotti cells (Jiang et al., 2015). They also receive a strong lateral projection from pyramidal cells in neighboring columns (Adesnik et al., 2012) and receive most of their excitation via NMDA receptors (Lu et al., 2007). The third type of interneuron expresses calretinin and vasoactive intestinal peptide (CR/VIP) (Tremblay et al., 2016) and targets CB/SST inhibitory neurons (Lee et al., 2013). Although gene expression of PV, SST and VIP have been used to successfully distinguish non-overlapping classes of interneurons in primates (Hodge et al., 2019; Krienen et al., 2020), in primates SST antibodies often label relatively few cells (Hendry et al., 1984; Mueller et al., 2018, 2020). SST is often, but not always co-expressed with CB (González-Albo et al., 2001; Lake et al., 2016). CB and SST expressing cells show a similar pattern of expression across cortical layers and areas in the macaque (Dienel et al., 2020). CR is expressed in most VIP neurons in primate cortex (Gabbott and Bacon, 1997; Lake et al., 2016), and both VIP and CR show a similar expression across layers and cortical areas in the macaque (Dienel et al., 2020). However, the investigation of cross-species interneuron type similarities and differences is ongoing and not resolved (Hodge et al., 2019; Kooijmans et al., 2020; Krienen et al., 2020). In our model, the three interneuron types should be more appropriately interpreted according to their synaptic targets, rather than other cellular markers.

See Table S6 for all parameter values.

#### Dopamine modulation

The density of dopamine D1 receptors per neuron was rescaled, so that the area with minimum density $\rho_{min}^{raw}$ was set to zero, and the area with maximum density $\rho_{max}^{raw}$ was set to one, with all other areas lying in between.

$$\rho_{\left[k\right]}=\frac{\rho_{\left[k\right]}^{raw}-\rho_{min}^{raw}}{\rho_{max}^{raw}-\rho_{min}^{raw}}$$

for all cortical areas *k*.

Network behavior was investigated for differing amounts of cortical dopamine availability (*λ*^*DA*^). The specific value of *λ*^*DA*^ used for each simulation is shown in the figures and main text. Note that for Figure 6, *λ^DA^* is calculated dynamically throughout each trial. Cortical dopamine availability is related to the fraction of occupied D1 receptors *λ*^*occ*^ through a sigmoid function. The fraction of occupied D1 receptors thus lies between 0 and 1, as expected.

(Equation 8) $$\lambda^{occ}=\frac{e^{b^{o}\left(\lambda^{DA}-c^{o}\right)}}{1+e^{b^{o}\left(\lambda^{DA}-c^{o}\right)}}$$

Dopamine increases the proportion of inhibition onto the dendrites of pyramidal cells (Gao et al., 2003). Therefore, we simulated the effect of dopamine on dendritic inhibition as follows. The total amount of dendritic inhibition increases (from a minimum to a maximum strength) as the total amount of occupied receptors increases. The total amount of occupied receptors is equal to the receptor density multiplied by the fraction of occupied receptors.

(Equation 9) $$g_{E_{dend},SST,[k]}^{DA}=g_{E_{dend},SST}^{min}+\lambda^{occ}\rho_{\left[k\right]}\left(g_{E_{dend},SST}^{max}-g_{E_{dend},SST}^{min}\right)$$

Dopamine decreases the proportion of inhibition onto the soma of pyramidal cells (Gao et al., 2003). Therefore, we simulated the effect of dopamine on somatic inhibition as follows. The total amount of somatic inhibition decreases (from a maximum to a minimum strength) as the total amount of occupied receptors increases.

(Equation 10) $$g_{E_{soma},PV,[k]}^{DA}=g_{E_{soma},PV}^{max}+\lambda^{occ}\rho_{\left[k\right]}\left(g_{E_{soma},PV}^{min}-g_{E_{soma},PV}^{max}\right)$$

Dopamine also increases the strength of excitatory synaptic transmission via NMDA receptors (Seamans et al., 2001). We modeled this with a sigmoid function, so that dopamine primarily increases NMDA conductances at low and medium dopamine concentrations, before reaching a plateau (Brunel and Wang, 2001).

(Equation 11) $$\nu_{\left[k\right]}=\frac{e^{b^{\nu }\left(\lambda^{occ}\rho_{\left[k\right]}-c^{\nu }\right)}}{1+e^{b^{\nu }\left(\lambda^{occ}\rho_{\left[k\right]}-c^{\nu }\right)}}$$

Here *b^v^* sets the slope of the sigmoid function, *c^v^* sets the midpoint.

The effects of dopamine on NMDA transmission is then defined as

(Equation 12) $$\nu_{\left[k\right]}^{DA}=1+\alpha \nu_{\left[k\right]}$$

where α controls the strength of dopamine modulation on NMDA transmission.

High levels of D1 agonism lead to a reduction in pyramidal cell firing, particularly during the delay period of working memory tasks. D1 receptor stimulation may lead to inhibition of ongoing activity by engaging an intracellular pathway involving cyclic AMP, protein kinase A and either HCN or KCNQ channels (Arnsten et al., 2019; Gamo et al., 2015; Vijayraghavan et al., 2007). The mechanisms by which HCN channels may hyperpolarise the cell are still under debate (George et al., 2009; Pereira, 2014). We simulated an increase in adaptation for very high levels of D1 receptor stimulation with a sigmoid function, so that adaptation increases at high dopamine concentrations, before reaching a plateau.

(Equation 13) $$\mu_{\left[k\right]}^{M}=\frac{e^{b^{M}\left(\lambda^{occ}\rho_{\left[k\right]}-c^{M}\right)}}{1+e^{b^{M}\left(\lambda^{occ}\rho_{\left[k\right]}-c^{M}\right)}}$$

#### Description of dynamical variables

The neural populations interact via synapses that contain NMDA, α-amino-3-hydroxy-5-methyl-4-isoxazolepropionic acid (AMPA) and gamma-aminobutyric acid (GABA) receptors. Each receptor has its own dynamics, governed by the following equations.

The synaptic variables are updated as follows (Wang 1999; Wong and Wang, 2006; Yang et al., 2016)

(Equation 14) $$\frac{ds^{NMDA}}{dt}=-\frac{s^{NMDA}}{\tau^{NMDA}}+(1-s^{NMDA})\gamma_{NMDA}r_{E}$$

(Equation 15) $$\frac{ds^{AMPA}}{dt}=-\frac{s^{AMPA}}{\tau^{AMPA}}+\gamma_{AMPA}r_{E}$$

(Equation 16) $$\frac{ds^{GABA}}{dt}=-\frac{s^{GABA}}{\tau^{GABA}}+\gamma_{l}r_{l}$$

(Equation 17) $$\frac{ds^{GABA,dend}}{dt}=-\frac{s^{GABA,dend}}{\tau^{GABA,dend}}+\gamma_{l}r_{l}$$

where *s* is the synaptic drive onto a particular receptor type, τ is the time constant of decay of that receptor and *γ*_*NMDA*_, *γ*_*AMPA*_ and *γ_I_* are constants. *r_E_* and *r*I** are the firing rates of the presynaptic excitatory and inhibitory cells targeting the NMDA, AMPA and GABA receptors, calculated below. Note that the inhibition onto the dendrite is slower than inhibition elsewhere (*τ*^*GABA,dend*^ > *τ*^*GABA*^) (Ali and Thomson, 2008). Hence we calculate dynamics of dendritic and somatic inhibition separately.

Adaptation acts to reduce the firing rate when the rate is high and has been frequently modeled in the following simple form (Engel and Wang, 2011; Hansel and Sompolinsky, 1998; Laing and Chow, 2002; Shpiro et al., 2007; Theodoni et al., 2011), derived from a spiking model (Liu and Wang, 2001; Theodoni et al., 2011)

(Equation 18) $$\frac{da}{dt}=-\frac{a}{\tau^{a}}+r$$

where *a* is the adaptation variable, *τ^a^* is the adaptation time constant, and *r* is the firing rate of the neural population.

#### NMDA/AMPA ratio

The fraction of excitatory postsynaptic current that is dependent on NMDA versus AMPA receptors differs by cell type (e.g., with relatively more current via the NMDA receptors in CB/SST versus PV cells) (Lu et al., 2007). Thus, we allowed the strength of excitatory transmission via NMDA and AMPA receptors to vary by cell type, described in the NMDA fraction, κ (Table S6).

#### Modulation of excitatory connections by dendritic spines

Approximately 90% of excitatory synapses on neocortical pyramidal cells are on dendritic spines (Nimchinsky et al., 2002). On this basis, we modulate the strength of excitatory connections according to the dendritic spine count.

$$\zeta_{\left[k\right]}=\frac{\zeta_{\left[k\right]}^{raw}-\zeta_{min}^{raw}}{\zeta_{max}^{raw}-\zeta_{min}^{raw}}$$

for all cortical areas [*k*].

(Equation 19) $$z_{\left[k\right]}=z^{min}+\zeta_{\left[k\right]}(1-z^{min})$$

where *z^min^* sets the lower bound for the modulation of excitatory connections by the spine count, *ζ*.

#### Description of local currents

The local NMDA current is calculated as follows

(Equation 20) $$I_{i,[k]}^{NMDA,local}=z_{\left[k\right]}\kappa_{i}\nu_{\left[k\right]}^{DA}\sum_{j\varepsilon \{E_{1},E_{2}\}}g_{i,j}^{E}s_{j}^{NMDA}$$

where the local excitatory connections via the NMDA receptors are scaled by the NMDA receptor fraction *κ_i_*, the dendritic spine count *z*_[*k*]_ and the D1 receptor stimulation $\nu_{\left[k\right]}^{DA}$ for all populations of neurons *i* and cortical areas *k*.

Similarly local excitatory connections via the AMPA receptors are scaled by the AMPA receptor fraction 1 − *κ_i_* and the dendritic spine count *z*_[*k*]_.

(Equation 21) $$I_{i,[k]}^{AMPA,local}=z_{\left[k\right]}\;(1-\kappa_{i})\sum_{j\varepsilon \{E_{1},E_{2}\}}g_{i,j}^{E}s_{j}^{AMPA}$$

Local inhibitory connections are not explicitly modulated by the dendritic spine count (as spines are the locations of synapses between excitatory cortical neurons). Note however, that the connectivity structure *g_GABA_* is modulated by the dopamine receptor density and occupancy (See Tables S2, S3, and S6).

(Equation 22) $$I_{i}^{GABA}=\sum_{j\varepsilon \{Inh\}}g_{i,j}^{GABA}s_{j}^{GABA}$$

where *Inh* is the set of inhibitory neuron populations.

The currents onto the dendrites are calculated separately, in order to calculate the nonlinear transformation of the current in the dendrite. They depend on the noise and background currents, so are described below.

#### Description of noise and background currents

Noise is modeled as an Ornstein-Uhlenbeck process, separately for each population.

(Equation 23) $$\tau^{AMPA}\frac{dI^{noise}(t)}{dt}=-I^{noise}(t)+\eta (t)\sqrt{\tau^{AMPA}\sigma_{noise}^{2}}$$

where *σ_noise_* is the standard deviation of the noise and η is Gaussian white noise with zero mean and unit variance.

A constant background current *I^bg^* was also added to each population (Table S6). This represents input from brain areas that are not explicitly modeled.

#### Description of the adaptation current

We include adaptation in excitatory cells (Kawaguchi, 1993), CB/SST (Kawaguchi, 1993, 1995) and CR/VIP cells (Mendonça et al., 2016; Schuman et al., 2019), but not PV cells (Kawaguchi 1993, 1995). This is reflected in their differing adaptation strengths $g_{PV}^{a}$ and $g_{other}^{a}$, where $g_{PV}^{a}=0$.

The adaptation current is

(Equation 24) $$I_{i,[k]}^{adapt}=\left(g_{i}^{a}+g_{i}^{m}\mu_{\left[k\right]}^{M}\right)a_{i,[k]}$$

for all local populations *i* and cortical areas *k*.

Note that $g_{i}^{a}$ represents the non-dopamine dependent adaptation, while $g_{i}^{m}\mu_{\left[k\right]}^{M}$ controls the dopamine-dependent adaptation, which depends on both dopamine release and receptor density (Equation 13).

#### Large-scale connectivity structure

Each of the cortical areas is connected using connectivity strengths derived from the retrograde tract-tracing data. Parts of this dataset of been included in previous publications (Markov et al., 2013, 2014a, 2014b). The long-range connectivity matrices are built from the FLN matrix. However, as noted in Mejias et al. (2016), the FLN matrix spans 5 orders of magnitude. The relationship between anatomical and physiological connectivity strengths is not clear, but if we were to use the raw FLN values in the large-scale model, many of the weaker connections would become irrelevant. To deal with this, we rescale the FLN matrix in order to increase the influence of smaller connections while maintaining the topological structure (Mejias et al., 2016; Mejias and Wang, 2021).

(Equation 25) $$w_{\left[k,l\right]}=\frac{FLN_{\left[k,l\right]}^{b_{1}}}{\sum_{l=1}^{n^{sub}}FLN_{\left[k,l\right]}^{b_{1}}}$$

Here we restrict calculations to the injected cortical areas *i*, *j*, which allows us to simulate the complete bidirectional connectivity structure within the subgraph (*n^sub^* = 40). We use the same parameter values as in Mejias et al. (2016) and Mejias and Wang (2021) (Table S6) to construct our interareal connectivity matrix *W*.

As noted previously, feedforward projections tend to originate in the supragranular layers, while feedback connections originate in the deep layers. Feedforward and feedback connections also likely have different cellular targets. Therefore it is useful to separate the long-distance feedforward and feedback connections.

(Equation 26) $$w_{\left[k,l\right]}^{supra}=SLN_{\left[k,l\right]}w_{\left[k,l\right]}$$

(Equation 27) $$w_{\left[k,l\right]}^{infra}=(1-SLN_{\left[k,l\right]})w_{\left[k,l\right]}$$

#### Interareal population interactions

The majority of interareal connections contain a mixture of axons projecting from deep and superficial layers. Long distance connections onto excitatory cells primarily target the distal dendrites (Petreanu et al., 2009; Table S4). Therefore, in the model we assume that long-distance connections target the dendrites of excitatory cells. CR/VIP cells receive the strongest long-distance inputs of all inhibitory cells, while CB/SST receives the weakest (Lee et al., 2013; Wall et al., 2016; Tables S5 and S6). This suggests that long-range connections effectively disinhibit the dendrite in the target area by exciting CR/VIP interneurons, while concurrently exciting the dendrite, to maximize the probability of information passing from the source area into the target area. Following Mejias and Wang (2021) we assume that feedback connections target inhibitory cells more strongly than feedforward connections.

Excitatory cells in different cortical areas with the same receptive fields are more likely to be functionally connected (Zandvakili and Kohn 2015). This is reflected in our model as follows. In the source area, there are two excitatory populations, 1 and 2, each sensitive to a particular feature of a visual stimulus (such as a location in the visual field). Likewise in the target area, there are two populations 1 and 2, sensitive to the same visual features. We assume that 90% of the output of population 1 in the source area goes to population 1 in the target area, and the remaining 10% to population 2. The converse is true for population 2 in the source area (it targets 10% population 1, 90% population 2; Tables S4 and S6).

#### Disinhibitory circuit in the frontal eye fields

The frontal eye fields (areas 8m and 8l in the model), have a very high percentage of calretinin neurons, and relatively fewer parvalbumin and calbindin neurons (Pouget et al., 2009). To account for this in the model, we relatively increased the long-range inputs to CR/VIP cells in areas 8m and 8l, as detailed in Table S6. These changes are critical for persistent activity in areas 8l and 8m, but otherwise do not greatly affect the behavior of the model. Without this change, the overlap between the simulated delay activity pattern and the experimental delay activity pattern (as in Figure 3A) is still extremely high (17/19 areas correct, chi-square = 12.31 p = 0.0004), and the activity pattern depends on both the long-range connectivity (p = 0.001), and D1 receptor distribution (p = 0.008), but not the spine count (p = 0.19), and lesions to areas 8l and 8m have a smaller effect on distributed persistent activity. All other results are unchanged. We also increased the relative strength of local CR/VIP connections and reduced the relative strength of local PV connections in FEF, but found that this had no effect on model behavior, so the simulations in the paper are presented without the local changes in FEF.

#### Calculation of long-range currents

Long-range interactions are applied as follows:

(Equation 28) $$I_{i[k]}^{NMDA,E,E}=z_{\left[k\right]}\mu^{E,E}\nu_{\left[k\right]}^{DA}\kappa_{i}\sum_{l=1}^{n^{sub}}w_{\left[k,l\right]}^{supra}\sum_{j\varepsilon \{E_{1},E_{2}\}}g_{i,j}^{E,E}S_{j[l]}^{NMDA}$$

where *z*_[*k*]_ is the dendritic spine count for area *k* (as defined above), *μ^E,E^* is the long-range connectivity strength onto excitatory cells (See Table S6), $\nu_{\left[k\right]}^{DA}$ is the degree of dopamine modulation of NMDA currents for area *k*, *κ_i_* is the NMDA/AMPA fraction for population *i*, *w*_[*k,l*]_ is the connection strength from area *l* to area *k*, $g_{i,j}^{E,E}$ sets the long-range strength from population *j* to population *i* (Tables S4 and S6) and $S_{j[l]}^{NMDA}$ is the synaptic NMDA drive from population *j* in source area *l*.

Similarly,

(Equation 29) $$I_{i[k]}^{NMDA,I,E}=z_{\left[k\right]}\mu^{I,E}\nu_{\left[k\right]}^{DA}\kappa_{i}\sum_{l=1}^{n^{sub}}w_{\left[k,l\right]}^{infra}\sum_{j\varepsilon \{E_{1},E_{2}\}}g_{i,j}^{I,E}S_{j[l]}^{NMDA}$$

(Tables S5 and S6).

The total long-range current via the NMDA receptors, is simply the concatenation of the two above terms *I^NMDA,E,E^* and *I^NMDA,I,E^*.

(Equation 30) $$I^{NMDA,LR}=\left(I^{NMDA,E,E},I^{NMDA,I,E}\right)$$

The long-range AMPA current is calculated similarly,

(Equation 31) $$I_{i[k]}^{AMPA,E,E}=z_{\left[k\right]}\mu^{E,E}(1-\kappa_{i})\sum_{l=1}^{n^{sub}}w_{\left[k,l\right]}^{supra}\sum_{j\varepsilon \{E_{1},E_{2}\}}g_{i,j}^{E,E}S_{j[l]}^{AMPA}$$

(Equation 32) $$I_{i[k]}^{AMPA,I,E}=z_{\left[k\right]}\mu^{I,E}(1-\kappa_{i})\sum_{l=1}^{n^{sub}}w_{\left[k,l\right]}^{infra}\sum_{j\varepsilon \{E_{1},E_{2}\}}g_{i,j}^{I,E}S_{j[l]}^{AMPA}$$

(Equation 33) $$I^{AMPA,LR}=\left(I^{AMPA,E,E},I^{AMPA,I,E}\right)$$

#### Description of dendritic currents

The inhibitory current onto the dendrite comes from CB/SST cells and is modulated by dopamine (Table S2; Equation 9)

(Equation 34) $$I_{i}^{dend,inh}=\sum_{j\varepsilon \{SST1,SST2\}}g_{i,j}^{GABA,dend}s_{j}^{GABA}$$

The distal dendrites receive long-range input (from neurons in other areas), noise and background input. In addition, if the area receives a stimulus directly, then the external stimulus also targets the dendrites. Note that most local connections target the area around the soma (Markram et al., 1997; Petreanu et al., 2009). This is reflected in the model by having local connections exclusively target the soma compartment of pyramidal cells.

(Equation 35) $$I_{i,[k]}^{dend,exc}=I_{i,[k]}^{NMDA,LR}+I_{i,[k]}^{AMPA,LR}+I_{i,[k]}^{stim}+I_{i,[k]}^{noise}+I_{i}^{background}$$

The dendritic nonlinearity is adapted from Yang et al. (2016) and modeled as follows:

(Equation 36) $$I^{soma,dend}=f_{l}\left(I^{dend,exc},I^{dend,inh}\right)=c_{1}.\left[\tanh\left(\frac{I^{dend,exc}+c_{3}I^{dend,inh}+c_{4}}{c_{5}e^{-I^{dend,inh}∕c6}}\right)\right]+c_{2}$$

where *I^soma,dend^* is the total current passed from the dendrite to the soma, *I^dend,exc^* and *I^dend,inh^* are the total excitatory and inhibitory current onto the dendrite, respectively. *c*1 to *c*6 control the gain, shift, inversion point and shape of the nonlinear function. These parameters are set to ensure that strong inhibition to the dendrite effectively blocks dendritic activity, but has little effect on somatic firing if the soma is directly stimulated (See Table S6; Marlin and Carter, 2014).

#### Application of external stimuli for tasks

In all simulations, the first stimulus is applied for 400ms. The second stimulus (Figures 3, 4, 5, and 6) is applied 600ms after the removal of the target stimulus for another 400ms. The two stimuli are of equal strength and duration, although the results are robust to a range of stimulus strengths (See Table S6 for parameter values). For Figures 2, 3, 4, 5, and 6 in the main text, a stimulus was applied to the dendrite of excitatory population 1 in area V1. For Figures 3, 4, 5, and 6 second stimulus was applied to the dendrite of excitatory population 2 of area V1. For Figures S4 and S5, the stimuli were applied to area 3 of primary somatosensory cortex instead. In all equations, the target and distractor stimuli are designated by the term *I^stim^*.

#### Total current in large-scale model

The total current equals the sum of all long-range, local and external inputs, and intrinsic currents.

(Equation 37) $$I_{total}=I^{NMDA,LR}+I^{AMPA,LR}+I^{NMDA,local}+I^{AMPA,local}+I^{GABA,local}+I^{soma,dend}+I^{adapt}+I^{noise}+I^{bg}+I^{stim}$$

#### Description of f-I curves

The f-I (current to frequency) curve of the excitatory population is

(Equation 38) $$f\left(I_{E}^{total}\right)=\frac{aI_{E}^{total}-b}{1-e^{-d\left(aI_{E}^{total}-b\right)}}$$

where *r_E_* is the firing rate of an populations of excitatory cells, $I_{E}^{total}$ is the total input to the population, *a* is a gain factor, *d* determines the shape of $f(I_{E}^{total})$, such that if *d* is large, $f(I_{E}^{total})$ acts like a threshold-linear function, with threshold *b* (Abbott and Chance, 2005).

The f-I curves for the inhibitory neuron populations are modeled using a threshold-linear function

(Equation 39) f(Iitotal)={ciIitotal+ri0forIitotal≥−ri0∕ci0,otherwise}

where *r_i_* is the firing rate of a population of inhibitory cells, $I_{i}^{total}$ is the total input to the population.

The threshold $r_{i}^{0}$ and slope *c_i_* depend on the cell type *i* (Bacci et al., 2003). See Table S6 for parameter values.

The firing rates are updated as follows

(Equation 40) $$\tau^{AMPA}\frac{dR}{dt}=-R+f\left(I^{total}\right)$$

for all cell types.

#### Short-term synaptic plasticity

For Figure 4, we added short-term plasticity to synapses from excitatory cells to excitatory cells (Hempel et al., 2000; Wang et al., 2004b) and CB/SST cells (Lee et al., 2013; Silberberg and Markram, 2007) as follows (Mongillo et al., 2008).

(Equation 41) $$\frac{ds^{NMDA}}{dt}=-\frac{s^{NMDA}}{\tau^{NMDA}}+xu(1-s^{NMDA})\gamma_{NMDA}\gamma_{xu}r_{E}$$

(Equation 42) $$\frac{ds^{AMPA}}{dt}=-\frac{s^{AMPA}}{\tau^{AMPA}}+xu\gamma_{AMPA}\gamma_{xu}r_{E}$$

(Equation 43) $$\frac{du}{dt}=\frac{U-u}{\tau^{u}}+U(1-u)r_{E}$$

(Equation 44) $$\frac{dx}{dt}=\frac{1-x}{\tau^{x}}-uxr_{E}$$

with *U* = 0.2, *τ^u^* = 1.5*s*, *τ*^*x*^ = 0.2*s*, as inMongillo et al. (2008). We also added a term *γ_xu_* = 2.5 to account for the fact that the product *xu* is usually less then 1, and to keep firing rates similar to those in other simulations.

#### Simulated transient inhibition of SST2 populations

In Figure 5, we simulate the effects of transient inhibition to the SST2 populations in cortical areas in the frontoparietal network. The frontoparietal network is defined according to the results of Leavitt et al. (2017), as in Figure 3. To do this, we apply an external inhibitory stimulus of 0.1nA to these populations for the duration of the distractor stimulus.

#### Dynamics and connectivity within VTA

For Figure 6, we investigate whether the dynamics of dopamine release can be learned in order to selectively maintain the desired working memory content. Previous cortico-basal ganglia models have tackled similar problems (Braver and Cohen, 2000; Frank, 2005). Note both dopaminergic and GABAergic cells in the VTA receive excitatory input from the cortex, while the majority of inhibition to dopaminergic cells comes from local VTA GABAergic cells (Soden et al., 2020).

The total current input to the dopamine cells in VTA is

$$I_{DA}^{tatal}=I_{DA}^{bg}+\sum_{k=1}^{n_{areas}}\sum_{j=1}^{2}c_{E_{j}}^{vta,ctx}g_{DA,E_{j}}^{vta,ctx}S_{NMDA,E_{j}}^{k}+\sum_{k=1}^{n_{areas}}\sum_{j=1}^{2}c_{E_{j}}^{vta,ctx}g_{DA,E_{j}}^{vta,ctx}S_{AMPA,E_{j}}^{k}+g_{DA,l}^{vta}S_{GABA}^{vta}$$

where $g_{DA,E_{j}}^{vta,ctx}$ sets the maximum strength of cortical-VTA connections. $c_{E_{j}}^{vta,ctx}$ is the fraction of synapses in an up state (Soltani and Wang, 2006), and is updated via reinforcement learning (see below). Initial values are $c_{1}^{vta,ctx}=0.7$, $c_{2}^{vta,ctx}=1$. $g_{DA,E_{j}}^{vta,ctx}=0.047nA$ $g_{DA,l}^{vta}=-0.55nA$, $I_{DA}^{bg}=0.35nA$.

The input to VTA inhibitory cells is

$$I_{vta,l}^{tatal}=I_{vta,l}^{bg}+\sum_{k=1}^{n_{areas}}\sum_{j=1}^{2}c_{E_{j}}^{vta,ctx}g_{l,E_{j}}^{vta,ctx}S_{NMDA,E_{j}}^{k}+\sum_{k=1}^{n_{areas}}\sum_{j=1}^{2}c_{E_{j}}^{vta,ctx}g_{l,E_{j}}^{vta,ctx}S_{AMPA,E_{j}}^{k}$$

where $g_{l,E_{j}}^{vta,ctx}=0.02nA$, $I_{vta,l}^{bg}=0.25nA$.

Synaptic inputs to the VTA inhibitory are driven by facilitating synapses (Soden et al., 2020), as in Equations 41-44, but with *x* = 0.87 held constant and *τ^u^* = 200*ms*.

The firing rates of the dopamine cells *r_DA_* are calculated as in Equations 38 and 40. The firing rates of GABAergic cells are updated as in Equations 39 and 40.

#### Cortical dopamine availability

Dopamine neurons fire bursts in response to stimuli that predict reward in working memory tasks (Schultz et al., 1993). Following release in the cortex, dopamine levels remain elevated for seconds (Muller et al., 2014). This is approximately the period of one trial in our simulations. Therefore, for the majority of simulations we approximated this by setting dopamine to a constant value for each trial.

For Figure 6 the cortical model is the same as in previous figures, with the exception that dopamine availability in the cortex *λ^DA^* changes dynamically and depends on the firing rates in the dopamine neurons, and *γ_NMDA_* = 6.41, *γ_AMPA_* = 25.

$$\frac{d\lambda^{DA}}{dt}=-\frac{\lambda^{DA}}{\tau^{DA}}+\gamma_{DA}r_{DA}$$

where *τ^DA^* = 2*s* and *γ_DA_* = 0.1. In addition, we removed the effect of dopamine on adaptation currents to simplify the learning process.

#### Reward-based learning

The fraction of cortex to VTA synapses in the up state is updated according to the outcome of the previous trial, using the simplified learning rule of Soltani and Wang (2006)

$$c_{E_{j}}(T+1)=c_{E_{j}}(T)+\alpha [1-c_{E_{j}}(T)]$$

if target *j* is selected and rewarded and

$$c_{E_{j}}(T+1)=c_{E_{j}}(T)-\alpha [c_{E_{j}}(T)]$$

if target *j* is selected and not rewarded. *T* is the current trial and *α* = 0.2 is the learning rate.

### QUANTIFICATION AND STATISTICAL ANALYSIS

#### Correlation between D1 receptor density and other anatomical features

Many aspects of brain anatomy are spatially autocorrelated, with nearby brain areas displaying similar anatomy. This spatial autocorrelation is not accounted for in conventional statistical tests, which often assume independence of data points. Failing to account for the spatial autocorrelation can lead to spurious correlations between brain maps. To overcome this problem, we generated random surrogate brain maps, with a spatial autocorrelation that closely matched the hierarchy map (Burt et al., 2020). This is done by first randomly permuting the values in the hierarchy map, and then smoothing and rescaling the permuted map to recover the lost spatial autocorrelation. The smoothing is perfomed by a local kernel-weighted sum of values of the *k* nearest neighbor regions, where *k* is chosen to best match the autocorrelation of the original hierarchy map (Burt et al., 2020). Each of the randomly generated surrogate maps is then correlated with the D1 receptor map. The spatially-corrected p value is then the fraction of surrogate maps that show a stronger Pearson correlation (negative or positive) with the D1 receptor map than the hierarchy map.

To compare the D1R density between granular and agranular cortical areas, we used a non-parametric Wilcoxon rank-sum test. To compare D1R density between areas with internopyramidisation, externopyramidisation and equal layer III and layer V pyramid sizes, we used a non-parametric Kruskall-Wallis test.

#### Comparing the simulated and experimental patterns of delay activity

In Figures 3A and 3B we compare the activity pattern of the model to the experimental pattern, and investigate its dependence on anatomical features. The experimental electrophysiology data was taken from a mega-analysis by Leavitt et al. (2017) of over 90 electrophysiology studies of delay period activity during working memory tasks. We first divided the cortex into persistent activity and non-persistent activity areas for both the experimental data and simulation (Table S7). Areas were classified in the persistent activity group if at least 3 more studies observed persistent delay period activity than a lack of such activity. We excluded areas that have been assessed in less than three studies. Of the areas that have been studied in at least three studies, we classify an area as having persistent activity, if more than 50% of studies have found persistent activity. However, the conclusions are not dependent on this threshold, or the minimum number of studies (Table S8). Areas in the simulation were classified as having persistent activity if, for the last 500ms of the trial, they had mean firing rates of at least 5Hz greater than the pre-stimulus baseline firing rates.

To shuffle anatomical connections, we shuffled connections within rows of the FLN matrix, so that the distribution of connections and connection strengths to each area remained constant, with the identity of the connections changing. The same reordering was applied to the SLN matrix. D1 receptor densities and spine counts were shuffled separately. Results were visualized using a custom version of a Raincloud Plot (Allen et al., 2019) to enable concurrent visualization of the distribution and individual simulation results. The p value is calculated as the fraction of simulations based on shuffled anatomical data that produce a delay activity pattern that overlaps with the experimental data as well as (or better than) the original simulation.

#### Lesioning of cortical areas

In Figures 3C-3H, we simulate the effects of a lesion to individual cortical areas. We do this by removing all input and output connections of the lesioned area in the connectivity matrices *W^E,E^* and *W^I,E^*. For the statistical analysis of the relationship between anatomical features and lesion effects, we removed areas V1 and V2 from the analysis. This was due to the fact that these areas were crucial to the propagation of the visual stimulus, but not working memory per se (Figure 3; Figure S5). We performed a stepwise-linear regression approach.

## Supplementary Material

1

2

3

4

5

6

7

8

## Figures and Tables

**Table:** KEY RESOURCES TABLE

REAGENT or RESOURCE	SOURCE	IDENTIFIER
Deposited data		
D1R/neuron data	This paper	BALSA:
40 area connectivity data	This paper	CORE-NETS:
Cortical representation of anatomical data	This paper	BALSA: https://balsa.wustl.edu/study/7qKNZ
Spine count data	Guy Elston (Elston, 2007)	https://doi.org/10.1016/B0-12-370878-8/00164-6
Neuron density data	Jon Kaas (Collins et al., 2010)	https://doi.org/10.1073/pnas.1010356107
Experimental models: Organisms/strains		
Cynomolgus macaque (*Macaca fascicularis*)	Labcorp (Covance)	https://drugdevelopment.labcorp.com/
Cynomolgus macaque (*Macaca fascicularis*)	Noveprim group, Ebene, Mauritius Camarney SL-Noveprim Europe, Camarles-Tarragona, Spain	http://www.noveprimgroup.com/
Rhesus macaque (*Macaca mulatta*)	Silabe, Centre de Primatologie Université Louis Pasteur, Strasbourg, France; Station de Primatologie de Rousset, Rousset-sur Arc, France	https://primatologie.unistra.fr/; http://www.celphedia.eu/en/centers/primatologie-rousset
Software and algorithms		
Large-scale dynamical model simulation and analysis software	This paper	Zenodo: https://doi.org/10.5281/zenodo.5507279
Python programming language	Python	RRID: SCR_008394
MATLAB 2019a	Mathworks	RRID: SCR_001622
BrainSMASH statistical testing of spatially autocorrelated brain maps	Burt et al. (2020)	https://github.com/murraylab/brainsmash
MATLAB Gifti toolbox	Guillaume Flandin	https://github.com/gllmflndn/gifti

## Highlights

- A gradient of dopamine receptors increases along the macaque cortical hierarchy
- A connectome-based dynamical model incorporates dopamine and multiple cell types
- Dopamine release switches from synaptic to persistent activity memory mechanisms
- Dopamine modulates dendritic inhibition to protect working memory from distractors
