# Supplementary figures and images for: A dopamine gradient controls access to distributed working memory in the large-scale monkey cortex
Source: Neuron. Author manuscript; Available in PMC 2021 Nov 6. (PMC8571070; doi:10.1016/j.neuron.2021.08.024)

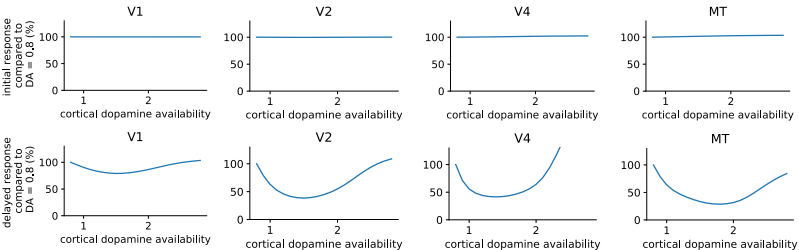

Supplement: 2 [file NIHMS1742228-supplement-2.pdf]

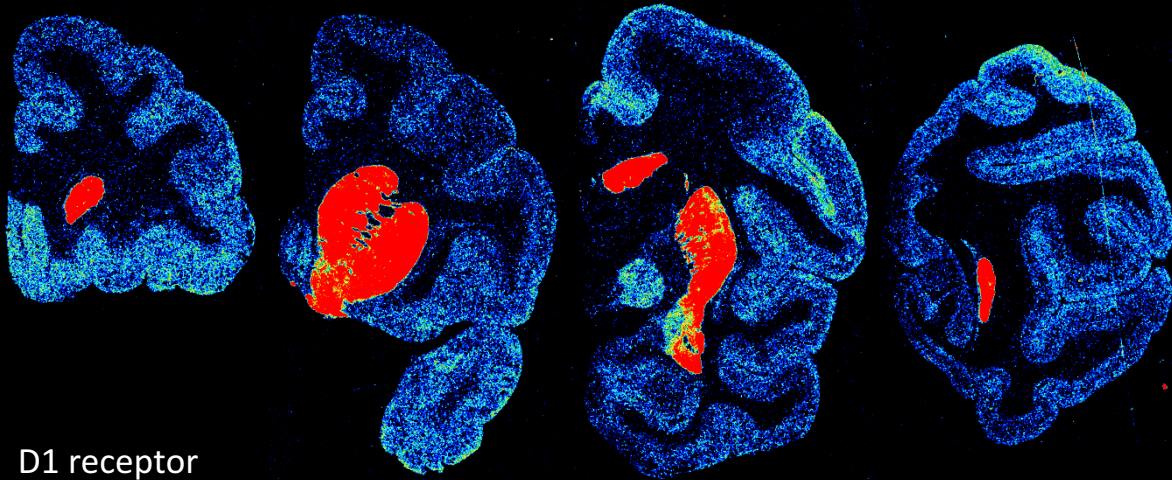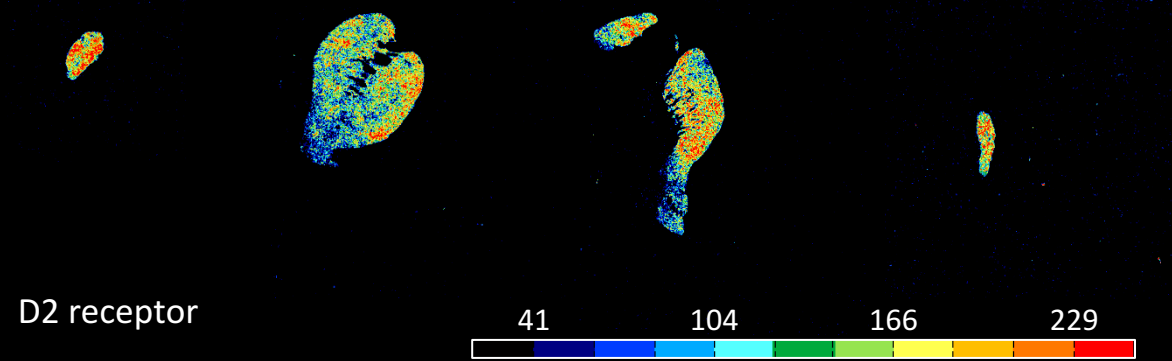

Supplement: 3 [file NIHMS1742228-supplement-3.pdf]

A)

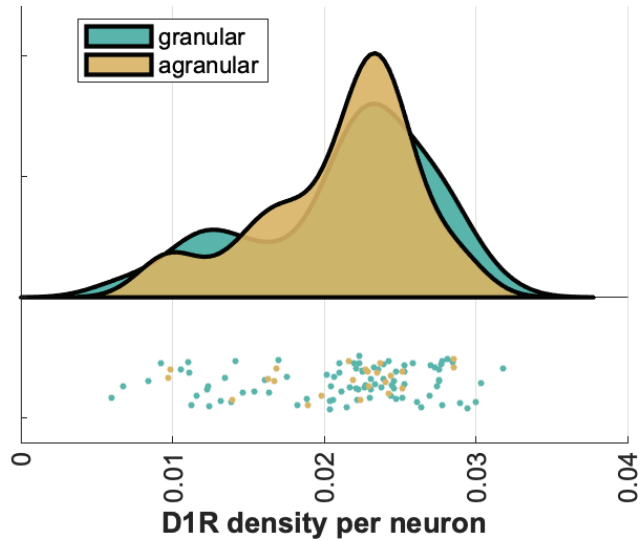

B)

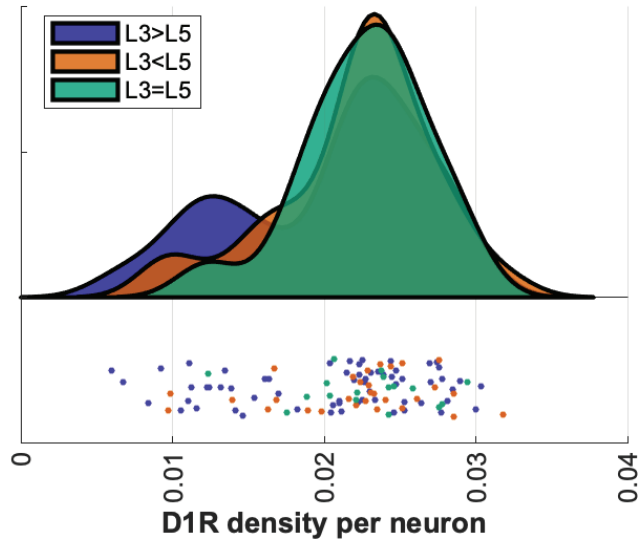

Supplement: 4 [file NIHMS1742228-supplement-4.pdf]

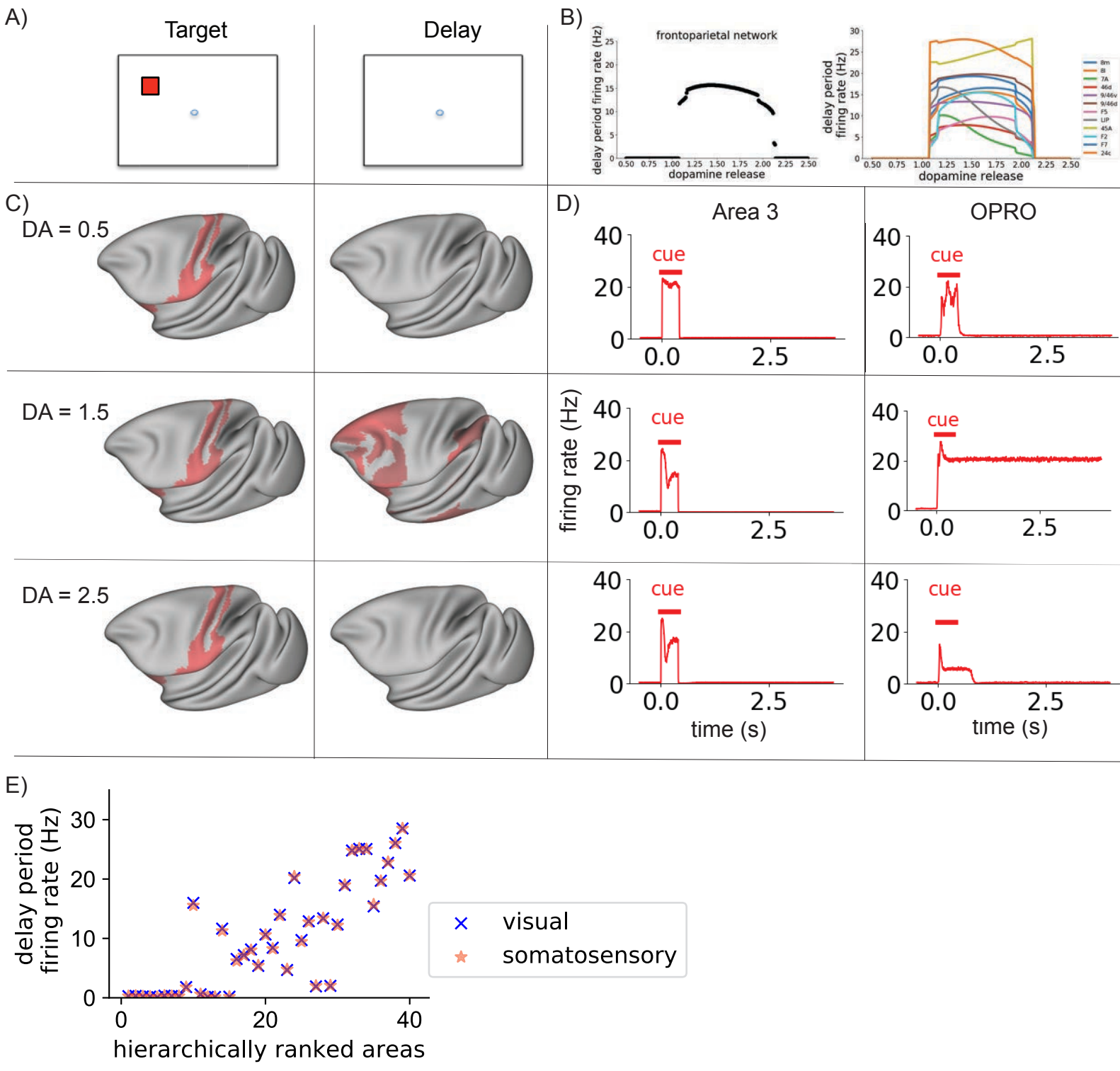

Supplement: 5 [file NIHMS1742228-supplement-5.pdf]

A)

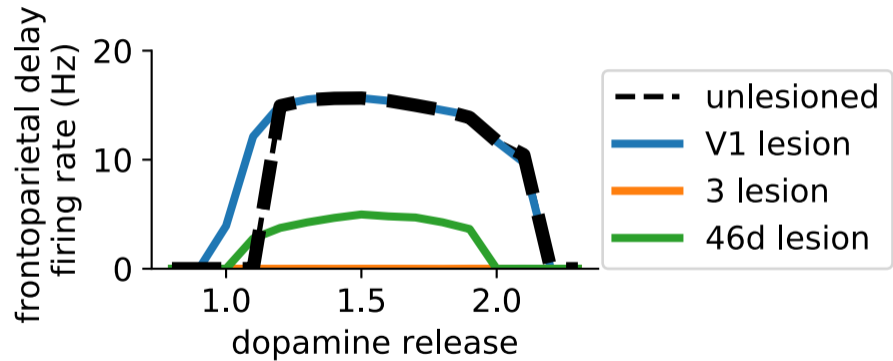

B)

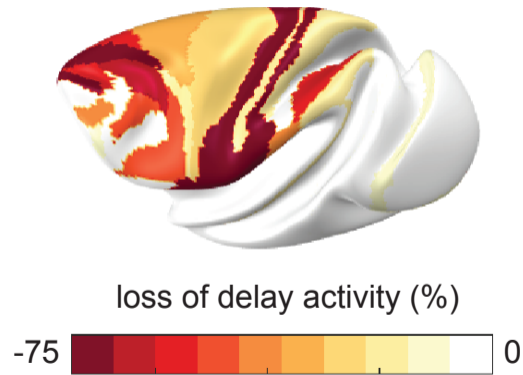

Supplement: 6 [file NIHMS1742228-supplement-6.pdf]
