# Supplementary material for: A dopamine gradient controls access to distributed working memory in the large-scale monkey cortex
Source: Neuron. Author manuscript; Available in PMC 2021 Nov 6. (PMC8571070; doi:10.1016/j.neuron.2021.08.024)
Supplement: 7 [file NIHMS1742228-supplement-7.pdf]

1 Supplementary Figures

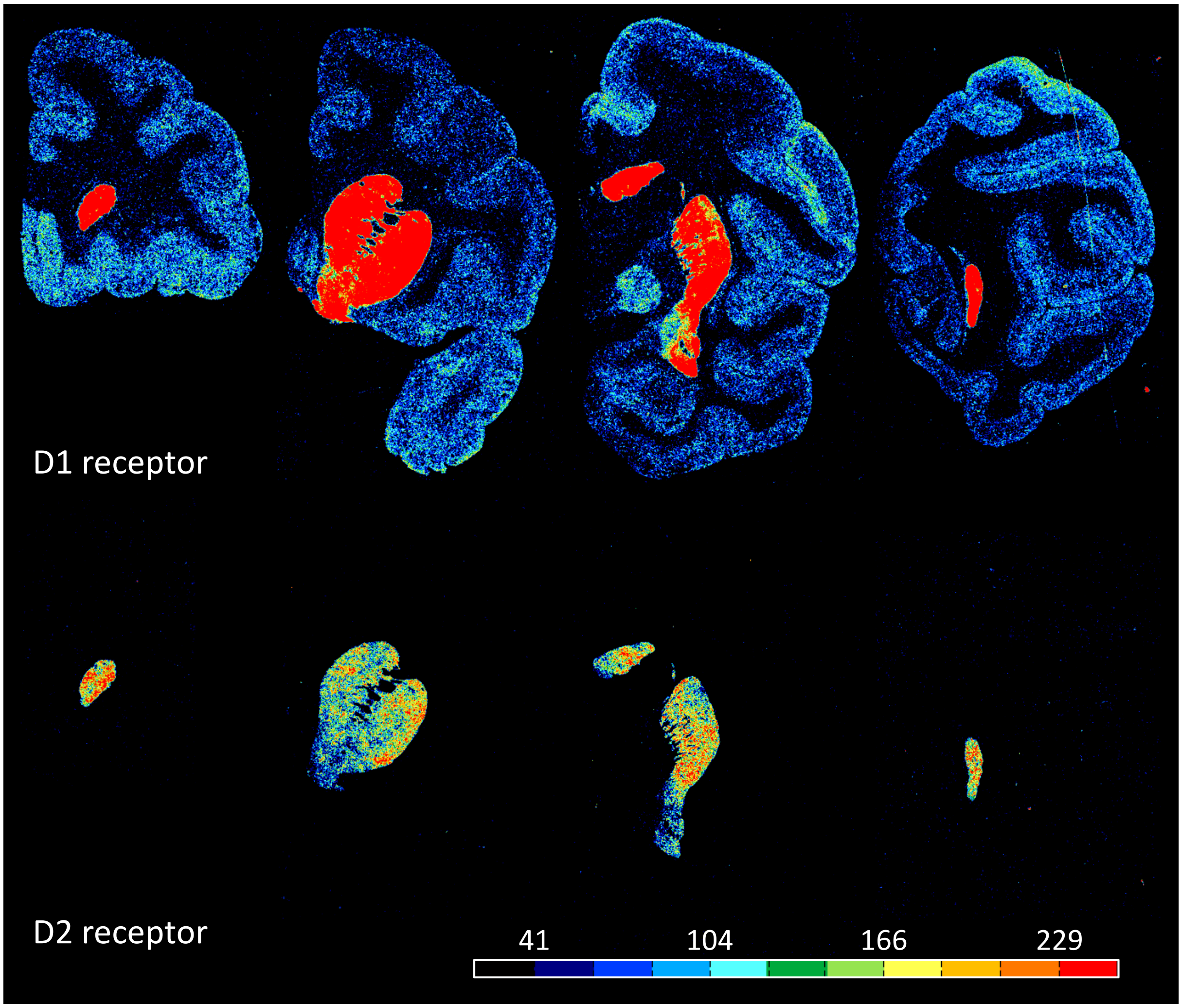

Figure S1: Example coronal sections through the macaque brain and processed for visualization of dopamine D1 and D2 receptors by means of quantitative in-vitro receptor autoradiography. Related to Figure 1. Note, that D2 receptor density in cortex is so low, that it is not detectable by means of the here applied method. Scale bar codes for receptor densities in fmol/mg protein.

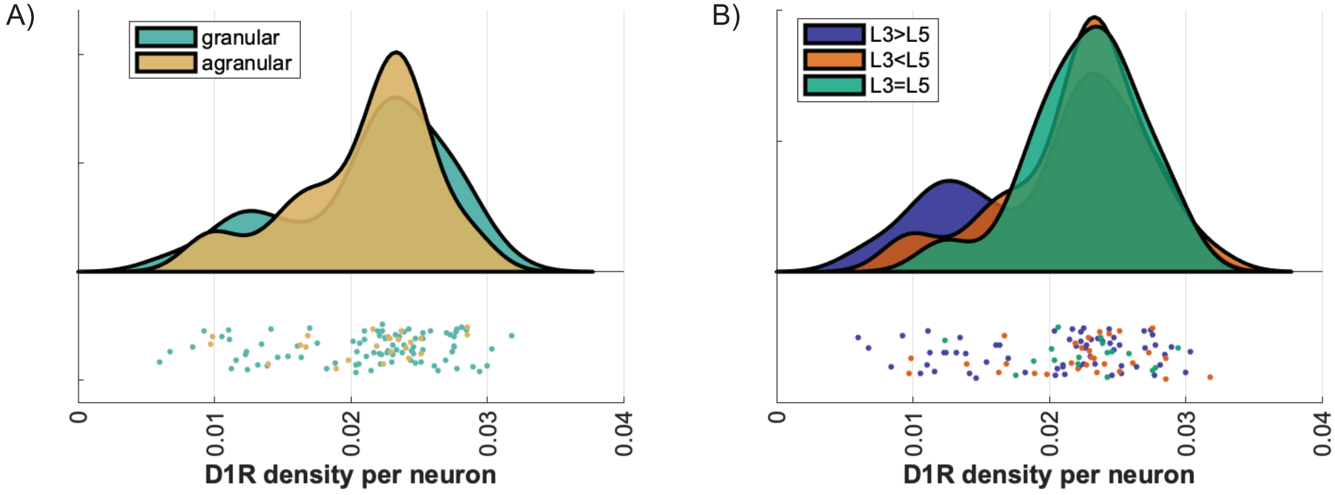

Figure S2: Anatomical distribution of D1 receptors. Related to Figure 1. A) The D1 receptor density per neuron did not significantly differ between granular (green) and agranular (yellow) areas. B) The D1 receptor density per neuron did not significantly differ between areas with larger pyramids in layer III (blue), those with larger pyramids in layer V (orange) or those with roughly equal sized pyramids in layers III and V (green)

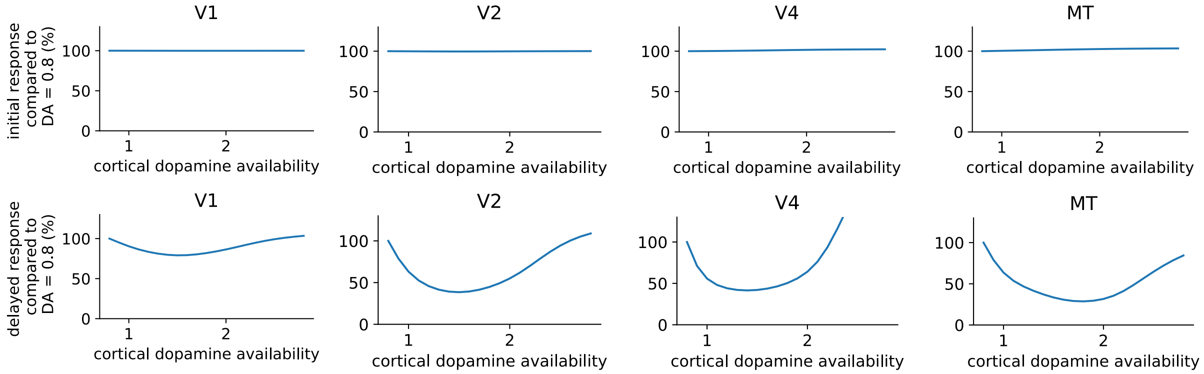

Figure S3: DA affects delayed, but not initial responses in early sensory areas. Related to Figure 2. Top row: The height of the initial peak response to the stimulus is essentially unaffected by dopamine levels in the cortex. Bottom row: The height of the delayed sensory response is affected by cortical dopamine levels, likely via feedback connections from higher cortical areas.

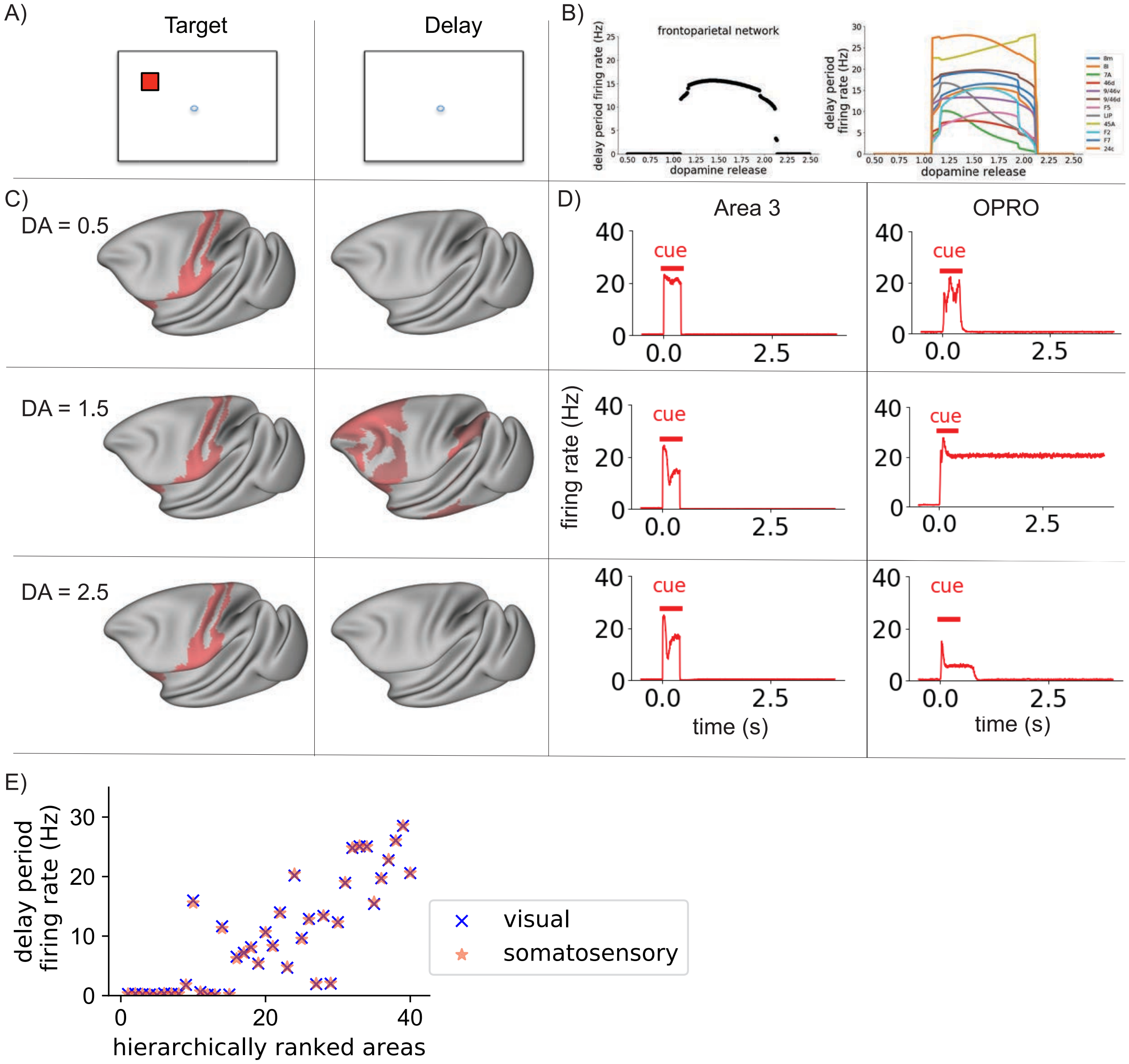

Figure S4: Dopamine release enables distributed somatosensory working memory. Related to Figure 2. A) Structure of the task. The cortical network was presented with a stimulus, which it had to maintain through a delay period. The tactile stimulus is presented to primary somatosensory cortex (area 3). B, left) Mean firing rate in the frontoparietal network at the end of the delay period, for different levels of dopamine release. There is an inverted-U relationship between dopamine release and delay period activity across the frontoparietal network, as for visual working memory. B, right) Mean delay-period activity of cortical areas as a function of dopamine release. All areas shown display persistent activity in experiments (Leavitt et al. 2017). C) Activity is shown across the cortex at different stages in the working memory task (left to right), with increasing levels of dopamine release (from top to bottom). Red represents activity in the excitatory population sensitive to the location of the target stimulus. Very low or very high levels of dopamine release resulted in reduced propagation of stimulus-related activity to frontal areas and a failure to engage persistent activity. Mid-level dopamine release enables distributed persistent activity. D) Timecourses of activity in selected cortical areas. The horizontal bars indicate the timing of cue (red) input to area 3. Activity in early somatosensory areas such as area 3 peaks in response to the stimulus, but quickly decays away after stimulus removal for all levels of dopamine release. In contrast, there is dopamine-dependent persistent activity in area OPRO. E) The pattern of activity at the end of the delay period is highly overlapping following visual and somatosensory working memory tasks. DA, cortical dopamine availability.

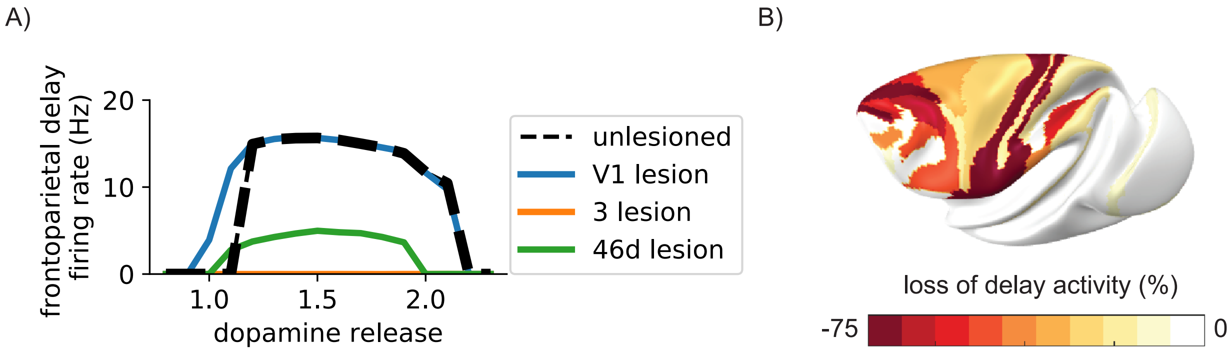

Figure S5: Lesions to visual areas do not disrupt somatosensory working memory. Related to Figure 3. A) Lesions to areas such as 46d and LIP led to reduced delay period firing across for all levels of dopamine release. Lesions to ares 3 and 2 of somatosensory cortex disrupted the ability to perform the somatosensory working memory task. In contrast, lesions to visual areas such as V1 did not significantly affect somatosensory working memory. B) Map showing the severity of lesions to cortical areas on somatosensory working memory. More severe effects are shown in deeper red.

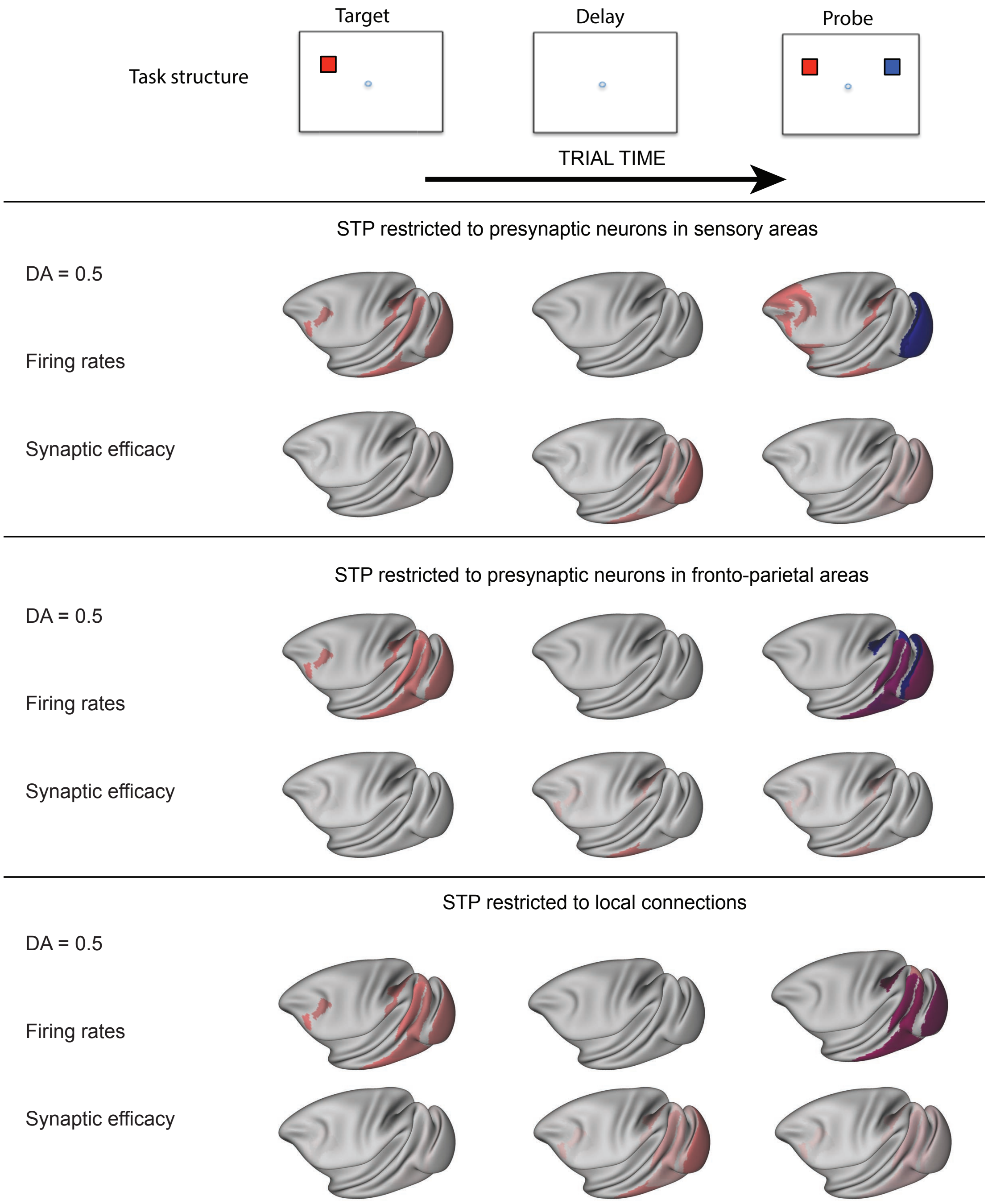

Figure S6: Activity-silent working memory without short-term plasticity in local prefrontal synapses. Related to Figure 4. Top row. The structure of the 'ping' short-term memory task. Second row. Reactivation of latent working memory representations was possible upon pinging the system, with short-term plasticity restricted to connections from neurons in sensory areas. Third row. Reactivation of latent working memory representations was not possible upon pinging the system, when short-term plasticity was restricted to connections from neurons in frontoparietal cortex. Bottom row. Reactivation of latent working memory representations was not possible upon pinging the system, when short-term plasticity was restricted to local connections between neurons in the same area.

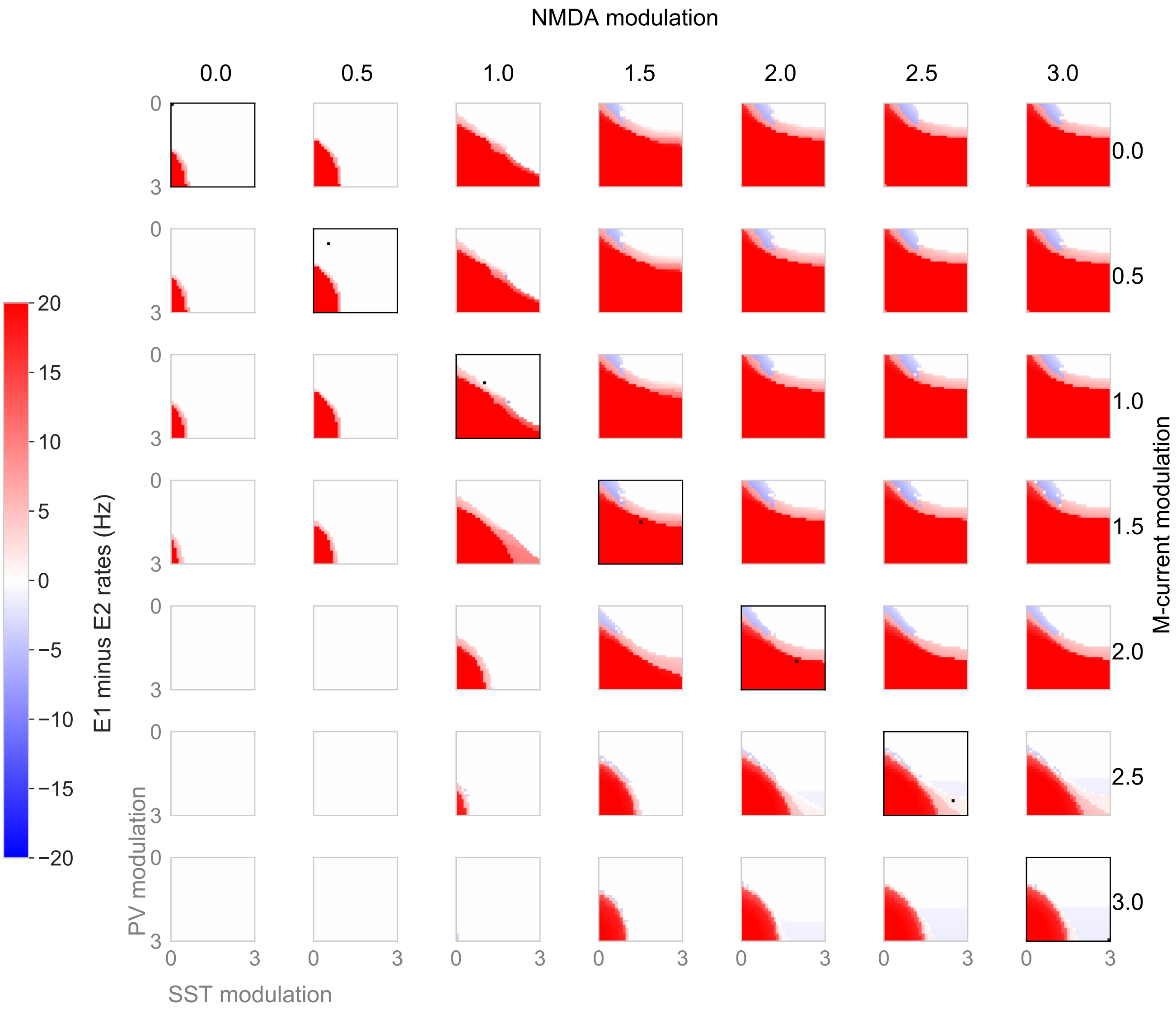

Figure S7: Distractor-resistance depends on the high dendritic inhibition. Related to Figure 5. We identified the model behaviour for different dopamine levels, across different levels of dendritic and somatic inhibition. Consistently across dopamine levels, higher somatic, and lower dendritic inhibition was associated with distractible working memory (blue). In contrast, lower somatic, and higher somatic inhibition was associated with distractor-resistant working memory (red). High dendritic and high somatic inhibition results in no persistant activity (white). The levels of dendritic and somatic inhibition associated with the standard dopamine modulation used in the rest of the paper marked by a black square. Note that high PV modulation by dopamine results in lower PV inhibition of the soma.

Supplementary Tables

$$
\begin{array}{cc}
 & \text{from} \\
G_E & \begin{array}{cc} E1^{soma} & E2^{soma} \end{array} \\
\begin{array}{c} E1^{soma} \\ E2^{soma} \\ PV \\ to\ CB1 \\ CB2 \\ CR1 \\ CR2 \end{array} &
\begin{pmatrix}
g_{E,E}^{self} & 0 \\
0 & g_{E,E}^{self} \\
g_{PV,E} & g_{PV,E} \\
g_{CB,E}^{self} & g_{CB,E}^{cross} \\
g_{CB,E}^{cross} & g_{CB,E}^{self} \\
g_{CR,E} & 0 \\
0 & g_{CR,E}
\end{pmatrix}
\end{array}
$$

Table S1. Related to STAR Methods. Local connectivity from excitatory cells. Local excitatory output connections target excitatory and inhibitory populations.

$$
\begin{array}{cc}
 & \text{from} \\
G_{I,[k]}^{dend} & \begin{array}{cc} CB1 & CB2 \end{array} \\
to\ \begin{array}{c} E1^{dend} \\ E2^{dend} \end{array} &
\begin{pmatrix}
g_{E^{dend},CB,[k]}^{DA} & 0 \\
0 & g_{E^{dend},CB,[k]}^{DA}
\end{pmatrix}
\end{array}
$$

Table S2. Related to STAR Methods. CB/SST cells target the dendrites of excitatory cells.

$$
\begin{array}{cc}
 & \text{from} \\
G_{I,[k]}^{soma} & \begin{array}{ccccc} PV & CB1 & CB2 & CR1 & CR2 \end{array} \\
\begin{array}{c} E1^{soma} \\ E2^{soma} \\ PV \\ to\ CB1 \\ CB2 \\ CR1 \\ CR2 \end{array} &
\begin{pmatrix}
g_{E_{soma},PV,[k]}^{DA} & 0 & 0 & 0 & 0 \\
g_{E_{soma},PV,[k]}^{DA} & 0 & 0 & 0 & 0 \\
g_{PV,PV} & g_{PV,CB} & g_{PV,CB} & 0 & 0 \\
0 & 0 & 0 & g_{CB,CR} & 0 \\
0 & 0 & 0 & 0 & g_{CB,CR} \\
0 & g_{CR,CB} & 0 & 0 & 0 \\
0 & 0 & g_{CR,CB} & 0 & 0
\end{pmatrix}
\end{array}
$$

Table S3. Related to STAR Methods. Local connectivity from inhibitory neuron cell types. PV cells inhibit the cell body of pyramidal cells, but are themselves inhibited by other PV cells and CB/SST cells. CB/SST cells and CR/VIP cells mutually inhibit each other.

$$
\begin{array}{cc}
 & \text{from} \\
J^{E,E} & \begin{array}{cc} E1_{soma} & E2_{soma} \end{array} \\
to\ \begin{array}{c} E1_{soma} \\ E2_{soma} \\ E1_{dend} \\ E2_{dend} \end{array} &
\begin{pmatrix}
0 & 0 \\
0 & 0 \\
g_{E,E}^{LR,self} & g_{E,E}^{LR,cross} \\
g_{E,E}^{LR,cross} & g_{E,E}^{LR,self}
\end{pmatrix}
\end{array}
$$

Table S4. Related to STAR Methods. Long-range targets onto excitatory cells

$$
\begin{array}{cc}
 & \text{from} \\
 & \begin{array}{ccc} J^{I,E} & E1_{soma} & E2_{soma} \end{array} \\
to & \begin{array}{c} PV \\ SST1 \\ SST2 \\ VIP1 \\ VIP2 \end{array}
\begin{pmatrix}
g^{LR}_{PV,E} & g^{LR}_{PV,E} \\
g^{LR}_{SST,E} & 0 \\
0 & g^{LR}_{SST,E} \\
g_{VIP,E} & 0 \\
0 & g_{VIP,E}
\end{pmatrix}
\end{array}
$$

Table S5. Related to STAR Methods. Long-range targets onto inhibitory cells

Table S6. Parameters for Numerical Simulations		
Parameter	Description	Value
$g_{E,E}^{self}$, $g_{PV,E}$, $g_{SST,E}^{self}$, $g_{SST,E}^{cross}$, $g_{VIP,E}$	Excitatory synaptic strengths	0.18nA, 0.174nA, 0.0435nA, 0.0435nA, 0.058nA
$g_{E_{soma},PV}^{min}$, $g_{E_{soma},PV}^{max}$, $g_{PV,PV}$	PV synaptic strengths	-0.001nA, -0.4nA, -0.18nA
$g_{E_{dend},SST}^{min}$, $g_{E_{dend},SST}^{max}$, $g_{PV,SST}$, $g_{VIP,SST}$	CB/SST synaptic strengths	-0.09nA, -0.11nA, -0.17nA, -0.1nA
$g_{SST,VIP}$	CR/VIP synaptic strengths	-0.05nA
τ^{NMDA}, τ^{AMPA}	E synaptic time constants	60ms,2ms
τ^{GABA}, $\tau^{GABA,dend}$	I synaptic time constants	5ms, 10ms
τ^{a}	adaptation time constant	100ms
γ_{NMDA}, γ_{AMPA}, γ_{I}	synaptic rise constants	1.282, 5, 2
κ_{PV}, κ_{other}	$\frac{NMDA}{NMDA+AMPA}$ fraction	0.8, 0.9
z^{min}	Min spine val	0.45
σ_{noise}	std. dev. of noise	0.005nA
$I_{E_{soma}}^{bg}$, $I_{i\epsilon Inh}^{bg}$, $I_{E_{dend}}^{bg}$	Background inputs	0.31nA, 0.30nA, 0.03nA
c_{1-6}	Dendrite parameters	0.12nA, 0.13624nA, 7, 0nA, 0.00964nA, 0.02nA
g_{PV}^{a}, g_{other}^{a}	Adaptation strength	0nA, -0.004nA
a, b, d	f-I curve (E)	0.135 Hz/nA, 54Hz, 0.308s
$c_{SST,VIP}$, $r_{SST,VIP}^{0}$	f-I curve (SST, VIP)	132Hz/nA, 33Hz
c_{PV}, r_{PV}^{0}	f-I curve (PV cells)	330Hz/nA, 95Hz
b_1	rescale FLN	0.3
$g_{E,E}^{LR,self}$, $g_{E,E}^{LR,cross}$	Long-range E targets	0.9, 0.1
$g_{PV,E}^{LR}$, $g_{SST,E}^{LR}$, $g_{VIP,E}^{LR}$	Long-range I targets	0.31, 0.22, 0.47
$g_{PV,E}^{LR,FEF}$, $g_{SST,E}^{LR,FEF}$, $g_{VIP,E}^{LR,FEF}$	Long-range I targets FEF	0.2, 0.1, 0.7
b^{o}, c^{o}	D1 occupancy	2, 1
b^{N}, c^{N}, α	DA-NMDA modulation	10, 0.35, 0.6
b^{M}, c^{M}, g_{E}^{m}, g_{I}^{m}	DA-M current	14, 0.85, -0.5, 0
$\mu^{E,E}$, $\mu^{I,E}$	Long-range connectivity	1.45, 2.24
I^{stim}	target/distractor stimulus	0.1nA (main figures), 0.2nA (Figs S4 and S5)

Table S6. Related to STAR Methods. Parameters for Numerical Simulations

Table S7. Experimental evidence for delay activity (from Leavitt et al., 2017)

Cortical area	Positive findings	Negative findings
V1	2	3
V2	1	0
V4	2	3
1	0	3
3	0	3
MT	0	5
V6	0	0
DP	0	0
TEO	0	0
8m	18	0
F4	1	0
5	1	0
2	1	2
8l	15	0
STPc	1	0
7A	6	1
10	0	1
F3	2	0
TEpd	2	2
46d	24	0
9/46v	31	2
PBr	0	0
9/46d	32	2
F5	3	0
7m	0	0
25	0	0
LIP	7	0
32	0	0
STPi	1	0
9	1	0
45A	6	0
8B	2	0
7b	0	0
F2	3	0
F7	6	0
ProM	0	0
STPr	1	0
24c	6	0
OPRO	0	0

Table S7. Experimental evidence for delay activity (from Leavitt et al., 2017), Related to Figure 3. Areas considered 'persistent activity areas' in Figure 3 are shown in a red background. Areas considered 'non-persistent activity areas' are shown in a blue background. Areas with insufficient studies are shown with a white background. Note that the overlap between the model and the experimental data is high regardless of the minimum number of studies chosen, and the threshold to decide which are 'persistent activity areas' - see Table S8.

Table S8. Overlap of simulated activity with experimental data (%)					
Thresholds	1 study	2 studies	3 studies	4 studies	5 studies
50%	95	95	93	100	100
60%	95	95	93	100	100
70%	95	95	93	100	100
80%	95	95	93	100	100
90%	90	89	86	92	90

Table S8. Overlap of simulated activity with experimental data (%), Related to Figure 3. The overlap between the simulated delay activity pattern (from the model based on real anatomy) and the experimental pattern was high, regardless of the criteria used. Two thresholds were used. First, a threshold based on the number of studies was used to determine which brain areas to compare between the experimental data and simulations. This 'number of studies' threshold is shown in the columns. Once the areas for comparison have been chosen, we must decide which areas have significant evidence for persistent activity. This is based on the 'percent threshold', shown here in the rows. For a percent threshold of 50%, any included areas for which more than 50% of studies have found persistent activity is counted as a 'persistent activity area'. The experimental persistent activity areas are then compared to the areas showing persistent activity in the model simulation. In Figure 3 in the main text, we use a number of studies threshold of 2 studies and 50% percent threshold.
